# A predictable conserved DNA base composition signature defines human core DNA replication origins

**DOI:** 10.1038/s41467-020-18527-0

**Published:** 2020-09-21

**Authors:** Ildem Akerman, Bahar Kasaai, Alina Bazarova, Pau Biak Sang, Isabelle Peiffer, Marie Artufel, Romain Derelle, Gabrielle Smith, Marta Rodriguez-Martinez, Manuela Romano, Sandrina Kinet, Peter Tino, Charles Theillet, Naomi Taylor, Benoit Ballester, Marcel Méchali

**Affiliations:** 1 Institute of Human Genetics, CNRS - University of Montpellier, Montpellier, France; 2 Institute of Metabolism and Systems Research (IMSR), University of Birmingham, Birmingham, UK; 3 Centre for Computational Biology (CCB), University of Birmingham, Birmingham, UK; 4 Institute for Biological Physics, University of Cologne, Cologne, Germany; 5 Aix-Marseille University, INSERM, TAGC, UMR S1090, Marseille, France; 6 Life and Environmental Sciences (LES), University of Birmingham, Birmingham, UK; 7 Institut de Génétique Moléculaire de Montpellier (IGMM), University of Montpellier, CNRS, Montpellier, France; 8 Institut de Recherche en Cancérologie de Montpellier (IRCM), Montpellier, France; 9 Pediatric Oncology Branch, NCI, CCR, NIH, Bethesda, MD USA

**Keywords:** Genome informatics, Origin firing

## Abstract

DNA replication initiates from multiple genomic locations called replication origins. In metazoa, DNA sequence elements involved in origin specification remain elusive. Here, we examine pluripotent, primary, differentiating, and immortalized human cells, and demonstrate that a class of origins, termed core origins, is shared by different cell types and host ~80% of all DNA replication initiation events in any cell population. We detect a shared G-rich DNA sequence signature that coincides with most core origins in both human and mouse genomes. Transcription and G-rich elements can independently associate with replication origin activity. Computational algorithms show that core origins can be predicted, based solely on DNA sequence patterns but not on consensus motifs. Our results demonstrate that, despite an attributed stochasticity, core origins are chosen from a limited pool of genomic regions. Immortalization through oncogenic gene expression, but not normal cellular differentiation, results in increased stochastic firing from heterochromatin and decreased origin density at TAD borders.

## Abstract (web summary)

In metazoan the DNA sequence elements characterizing origin specification are unknown. By generating and analysing 19 SNS-seq datasets from different human cell types, the authors reveal a class and features of Core origins of replication which can be predicted by an algorithm.

## Introduction

During each cell division, a human cell will replicate ~2 m of DNA within the S-phase time constraints. To achieve this, DNA replication initiates from thousands of regions that are called DNA replication origins and are spread across the genome. The positioning of DNA replication initiation sites (IS) in the genome (origin specification) is poorly understood in metazoans. In prokaryotes and viruses, usually a single, sequence-specific origin exists, while in the eukaryote *Saccharomyces cerevisiae*, DNA replication initiates from AT-rich consensus sequences that are bound by the yeast origin recognition complex (ORC)^1^. By contrast, in fruit fly and mouse cells, the presence of a G-rich DNA sequence element, (Origin G-rich Repeated Element, OGRE), 300 bp upstream of the IS has been reported in more than 60% of origins^2–7^. CA/GT-rich motifs^2^ and poly-A/T tracks^8^ have also been detected at IS in mouse cells. OGRE elements may contain CpG islands (CpGi)^4,9–12^ and potential G-quadruplex (G4) elements^3,13,14^, in a nucleosome-free region^2^. However, only a fraction of all putative G4 elements in the genome host a nearby origin, and CpGi are present only in a fraction of origins. This indicates that other features contribute to replication origin selection or activation. Here, we identify origins in human cells using a Short Nascent Strand isolation protocol^4,10,13,15–17^ coupled with next-generation sequencing (SNS-seq) that minimises the false-positive rates (FPR; see Methods section). This allows characterisation of human DNA replication origins in human stem cells and during normal cellular differentiation, as well as after immortalisation upon oncogene mis-expression. This lets us define a subset of origins, which we call core origins, from which 80% of replication initiation takes place in all the tested human cell types. We also show that origin activity can be modulated by the transcriptional landscape. Although, we do not detect strict consensus sequences, we were able to predict the position of most core origins in the human and mouse genomes using computational algorithms based only on the DNA sequence. Cell immortalisation results in an increased stochasticity of origin positioning, especially in heterochromatin regions, as well as an alteration of their distribution along TAD domains.

## Results

### The landscape of DNA replication origins in the human genome

**Fig. 1:**
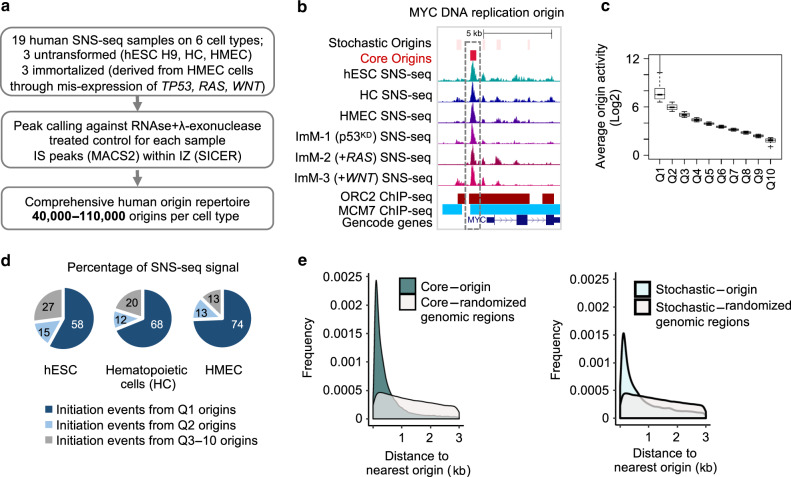
Human origin repertoire. **a** Experimental workflow. SNS-seq was performed on three untransformed (hESC H9, patient derived hematopoietic cells (HC), and patient derived Human Mammary Epithelial Cells (HMEC), and three immortalised cell types (total *n* = 19). Immortalised cells were obtained through a reduction of *TP53* mRNA levels (ImM-1, p53^KD^) or further expression of oncogenes *RAS* (ImM-2, +RAS) or *WNT* (ImM-3, +WNT) in HMEC cells. **b** UCSC genome browser snapshot of the human replication origin (*MYC* origin) captured by SNS-seq. Representative SNS-seq read-profiles, published positions of ORC2- (red) and MCM7-bound (blue) regions and the GENCODE genes (v25) are shown. The positions of origins defined in this study are shown on top; red: high-activity origins (core origins), light pink: low-activity origins (stochastic origins). **c** Boxplot showing the average origin activity (normalised SNS-seq counts across all samples, in Log2) per each quantile (*x*-axis represents Q1-Q10 origins). Line within the boxplot represents median, whereas the bounds of the box define the first and third quartiles. Bottom and top of whiskers represent minimum and maximum numbers respectively for each boxplot. **d** Q1 and Q2 origins host the overwhelming majority of initiation events in untransformed cell types. Pie chart representing the percentage of DNA replication initiation events (normalised SNS-seq counts) that originate from Q1, Q2 or Q3-10 origins in the indicated untransformed cell types. **e** Density plots showing the distribution of the distances to nearest origin (*x*-axis, in Kb) for core origins (left panel) and stochastic origins (right panel). In grey are control density plots that show the distribution of the distances between core/stochastic origins to the nearest randomised genomic region of the same size and number as origins. Both frequency plots were significantly different from randomised distributions (*p* ≤ 2.2E-16, Chi-square Goodness-of-Fit test in R with observed and expected values for frequency).

Using an optimised SNS-seq protocol (see Methods section and Supplementary Fig. 1a), we identified DNA replication IS from 19 human cell samples, representing three untransformed (human embryonic stem cells, hESC; cord blood CD34(+) hematopoietic cells, HC; primary human mammary epithelial cells, HMEC) and three immortalised cell types derived from the HMEC line (ImM-1, ImM-2, and ImM-3; Fig. 1a). Owing to the high number of cell samples investigated, a total of 320,748 IS were identified, the overwhelming majority of which were low-activity IS belonging to immortalised cell types (Supplementary Data 1a, b, see following section). The IS repertoire included the previously identified human LaminB2^18^, MYC^19^, MCM4^20^ and HSP70^21^ origins (Fig. 1b and Supplementary Data 1c). As the raw data clearly exhibited variations in replication origin activity, we classified origins in 10 quantiles, based on their average activity (i.e., mean normalised SNS-seq signal): from quantile 1 (Q1) that contained the top 10% (highest average activity) to quantile 10 (Q10) that included the bottom 10% (lowest average activity) of origins (Fig. 1c, Supplementary Fig. 1b). Origins in each quantile displayed similar mappability, which is a measure of the ability of SNS-seq reads to be matched to the human genome. Therefore, the variation in SNS-seq signal at origins belonging to different quantiles were not due to the technical differences in our ability to map them (Supplementary Fig. 1c).

Strikingly, our classification revealed that 70–85% of the origin SNS-seq signal originated from Q1 and Q2 origins in all cell types analysed (Fig. 1d and Supplementary Data 1b). In addition, we observe that almost all the enrichment of the SNS-seq signal across the genome comes from regions that are defined as origins in our study, suggesting that broad and diffuse initiation outside origin regions is not substantial (Supplementary Fig. 1d, see Methods section). As the SNS-seq signal represents the amount of DNA replication initiation events that take place in a cell population, we concluded that Q1 and Q2 origins host the majority of the initiation events, highlighting these 64,148 regions, termed “core origins”, as replication initiation hotspots, irrespective of the cell type.

The remaining 80% of IS (Q3–Q10, 256,600 regions), hereby termed “stochastic origins”, had low mean activity across 19 samples and only hosted ~15–30% of total SNS-seq signal in each cell type (Fig. 1d and Supplementary Data 1b).

Most core origins were clustered together, because the distance to the nearest origin was shorter for core origins compared with stochastic origins or random distribution (Fig. 1e and Supplementary Fig. 1b, e). This is consistent with a previously observed community effect whereby clustered origins have higher activity than isolated origins^4,10,22^ (Supplementary Fig. 1e). Remarkably, a similar number of core origins in *Mus musculus* host 69% of all initiation events detectable by SNS-seq, suggesting that the core origins are a feature not specific to the human genome (Supplementary Fig. 1f).

### The position of core origins is consistent

**Fig. 2:**
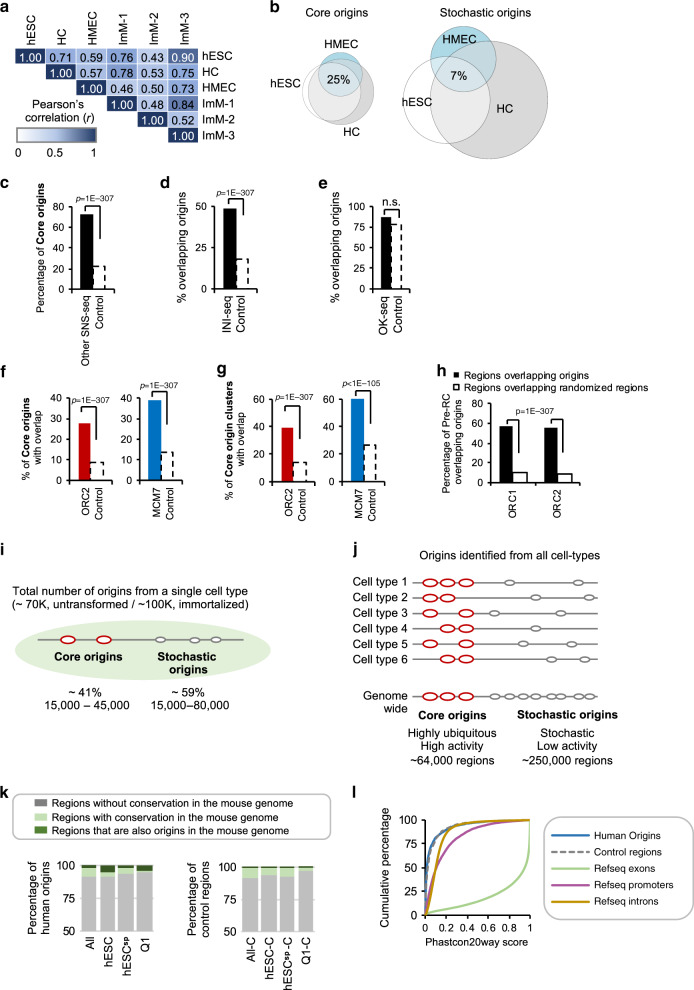
Higher activity origins are ubiquitously present across replicates and cell types. **a** Pearson’s correlation coefficient (*r*) of origin activities between cell types. **b** Euler diagrams showing the fraction of core and stochastic origins shared by the untransformed cell types. **c** Bar plots show the percentage of core origins that were identified as origin regions by another SNS-seq study^12^ (black), and the expected amount of overlap with control regions (white, dotted line). Control regions in this figure are regions of equal size to core origins that are located in randomised coordinates of the human genome. *P*-value obtained by Chi-square Goodness-of-Fit test. **d** Bar plot representing the percentage of regions identified by INI-seq^7^ (in black) that overlap origins identified in this study. Dotted bar represents the expected amount of overlap with control regions. *P*-value obtained by Chi-square Goodness-of-Fit test. **e** As in **d** for OK-seq^23^ regions. **f** Percentage of core origins that overlap with pre-RC components ORC2 (within ± 2Kb; in red) and MCM7 (direct overlap, in blue). Dotted bars represent the expected amount of overlap with control regions. *P*-values obtained by Chi-square Goodness-of-Fit test. **g** As in **f** for core origins found in clusters. **h** Bar plots show the percentage of ORC1- (~13,000) and ORC2-bound (~55,000) sites that host DNA replication initiation within 2 Kb. Dotted bars represent overlap with control regions. *P*-values obtained by Chi-square Goodness-of-Fit test. **i** Schematic summary of origin activity in a single cell type. **j** Schematic summary of origin activity in the different cell types. **k** Bar plots showing the percentage of all, hESC, hESC-specific, and Q1 human origins with homology to mouse (light green). Also indicated are regions in the human genome with a homologous region in the mouse (light green). Regions that are also origins in mouse are dark green. On the right, are bar plots showing the percentage of the corresponding shuffled genomic regions. **l** Cumulative Phastcon20way scores plotted for human DNA replication initiation sites (blue), similar-sized control regions (dotted, grey), Refseq exons (green), promoters (defined as 500 bp upstream of TSS regions, in purple) and introns (mustard).

Origin activity was highly correlated in the different cell types (Fig. 2a, average Pearson’s *r* = 0.69, *P*-value <2E-16 for all comparisons), suggesting that a given origin has similar levels of initiation in different cell types. About 77% of origins shared by the different cell types were core origins (Supplementary Data 1b). Conversely, stochastic origins were less shared (Fig. 2b and Supplementary Fig. 2a). In support of our findings that core origins are more ubiquitously active in different cell types, 72% of core origins were identified by an independent SNS-seq study^12^ using different cell types (Fig. 2c and Supplementary Fig. 2b). Moreover, 49% of regions identified by a different origin mapping method (INI-seq^7^) in a different cell line overlapped our origins, majority of which were core origins (Fig. 2d). Early firing core origins were more likely to be identified by INI-seq, which maps early firing origins (Supplementary Fig. 2c). In addition, almost all (87%) regions identified by OK-seq^23^, overlapped origins identified in this study (Fig. 2e). However, as this method only maps 5000–10,000  bp regions, with an average size of 34 kb; this overlap was not statistically significant. Nevertheless, core origins and core origins found in tight clusters (see Methods), which resemble initiation zones similar in size to those identified by OK-seq, overlapped significantly with regions identified by OK-seq (49.7%, Supplementary Fig. 2d, e).

Core origins also coincided with regions previously shown to be bound by the pre-replication complex (pre-RC) components ORC1^24^, ORC2^25^ and MCM7^26^. Specifically, 28% and 39% of core origins overlapped with ORC2 or MCM7-bound regions (Fig. 2f and Supplementary Fig. 2f). Clustered core origins (initiation zones) overlapped with pre-RC component-bound regions more often (40% with ORC2 and 60% with MCM7, Fig. 2g). Given that only about half of all core origins is active in any one cell type, the amount of overlap is suggestive that most active core origins are associated with pre-RC components ORC2 and MCM7. Reciprocally, 57% of ORC1- and 55% of ORC2-bound regions overlapped at least with one origin identified by SNS-seq (Fig. 2h). Broader ORC1- or ORC2-bound regions, which might represent regions with multiple ORC1/2 binding events as suggested in *S*. *pombe*^27^, were more likely to host an origin, and mostly a core origin (Supplementary Fig. 2g, h).

In summary, our analysis identified core origins that represent bona fide IS in different cell types, which are also identified by alternative origin mapping methods. On average, core origins represent ~40% of all origins identified in a single cell type, representing on average ~30,000 regions (Fig. 2i). It is worth noting that core origins are different from “constitutive/common origins” previously observed with SNS-seq data^12,13^. Our analysis has the highest number of samples amongst these studies and based on our data, we infrequently observe origins that are active in every sample (Supplementary Data 1d).

### Human and mouse genomes share a G-rich sequence signature

We next investigated whether DNA replication initiation sites are placed in homologous regions across mouse and human genomes. We find that only a small fraction (8%) of human origins have homologous regions in the mouse genome and only 2% are also identified as origins in mouse cells (Fig. 2k, left panel). We find a comparable level of homology for randomised genomic regions (7% conserved, 0.8% overlapping mouse origins, Fig. 2k, right panel) suggesting that the majority of DNA replication initiation sites are not located in homologous regions in the mouse and human genomes. In accordance, we observed a low level of sequence conservation of the origin DNA sequence compared to promoters and exonic regions across 20 mammalian species, reinforcing the idea that these sequences have appeared independently in the different lineages during evolution (Fig. 2l). Interestingly, Phastcon20way scores of regions flanking the origins (±5 Kb of origin summits), display moderately conserved regions 0.5–3 Kb upstream of the IS region for core origins, which are mostly attributable to regulatory elements/exonic sequences (Supplementary Fig. 2i, j).

**Fig. 3:**
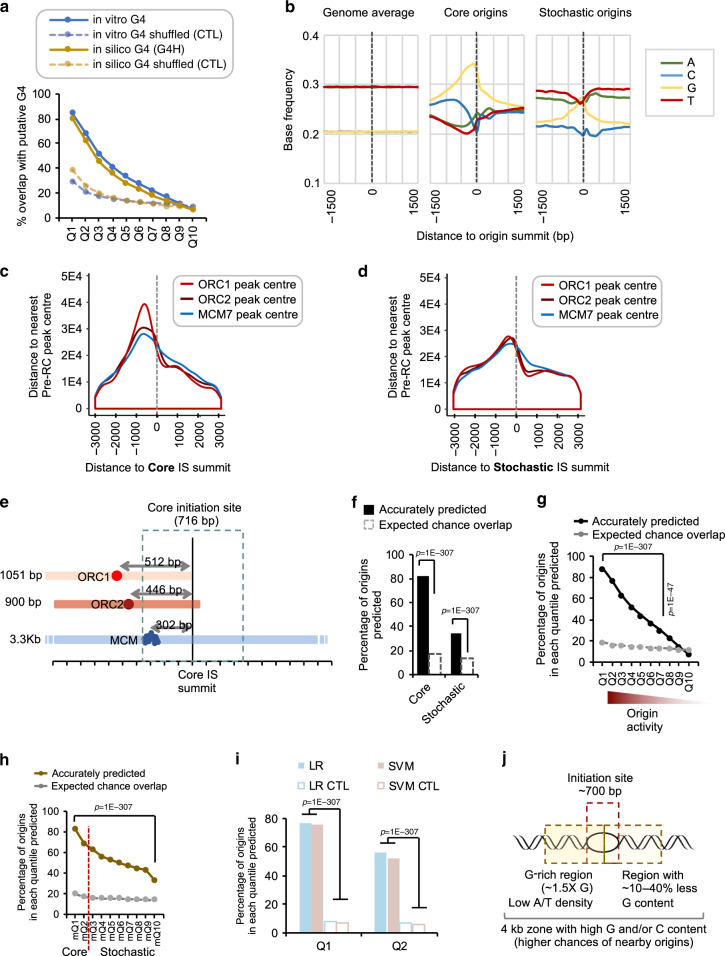
The DNA sequence content is a major predictor of DNA replication IS. **a** Graph showing the percentage of origins in each quantile that overlap with G4 defined by G4Hunter^29^ (in silico) or mismatches^28^ (in vitro G4). Dotted lines (CTL) represent overlap with control regions. **b** Base content of the regions flanking human DNA replication origins and control genomic regions. Frequency plots are centred at the origin summits. The base frequency represents the proportion of each base (0–1). The human genome is composed of 30% A,T and 20% G, C as indicated by genomic average. Origins are oriented with the highest G-content upstream. **c** Density plot represents the frequency of the distance measured between the initiation site summit (dotted line) and the centre /summit of the nearest ORC1 (red), ORC2 (dark red) and MCM7 (blue) bound regions. Origins are oriented with the highest G-content upstream. **d** As in **c** but for stochastic origins. **e** Schematic representation of a core origin. The vertical line represents the IS summit. The nearest ORC1, ORC2 and MCM7 peak centres are presented, as well as their average distance from the core IS summit. The average size of the ORC1, ORC2 and MCM7 binding sites is indicated on the left. **f** Bar plot showing the percentage of origins that can be predicted based on the genome-scanning (GS) algorithm. Dotted bars represent the expected amount of overlap with control regions. The pie chart shows the percentage of false-positive results (grey). *P*-values obtained by Chi-square Goodness-of-Fit test using observed and expected values for overlap. **g** Percentage of origins in each quantile predictable by the GS algorithm as in **f**. **h** Percentage of *Mus musculus* origins predicted by the GS algorithm as in **f**. **i** Bar plots representing the percentage of core origins that can be predicted using a combination of GS algorithm and two different machine-learning algorithms (single vector machine (SVM) and logistic regression (LR) with greedy feature selection). *P*-values obtained by Chi-square Goodness-of-Fit test using observed and expected values for overlap. **j** Schematic showing the properties of the regions predicted to be origins. G-richness in the immediate (0.5 Kb) and distal (2 Kb) upstream region to the initiation site are predictive parameters.

Despite lacking sequence homology, functional regions of the genome may contain sequence elements that are shared between species. Thus, we next examined sequence elements that might be shared across replication origins of different species. To identify DNA sequence elements that coincide with origins, we examined the relationship between the IS and G-rich putative G4 structures, which are helical DNA configurations that contain one or more guanine tetrads. 83% of core and 34% of stochastic origins contained at least one putative G4 element defined by two different methods^28,29^ (Fig. 3a and Supplementary Fig. 3a). A large number of putative G4 elements has been predicted in human and mouse genomes, but as previously noted, only a fraction of them hosts an origin^2–4,12^. Hence, the presence of a putative G4 element is not, on its own, a strong predictor of origin placement, but most core origins indeed contain a G4 element.

Similar to previous findings in mouse^2^, a number of G-rich motifs upstream of the IS were evident (Supplementary 3b) and were enriched in origin sequences even after C/G and CpG content normalisation of the control regions (Supplementary 3c). Analysis of the base composition of human origins within ±1.5 Kb of the oriented IS summit confirmed that core origins were enriched in G-rich sequences with an asymmetrical enrichment up to 1.5 Kb upstream of the IS centre (Fig. 3b).

We further asked how the replication origins determined in this study position relative to the placement of pre-RC factors on the genome. When we aligned the positions of the pre-RC components ORC1^24^, ORC2^25^ and MCM7^26^ relative to the IS, we found that they were preferentially positioned upstream of the IS, near the G-rich region in both core and stochastic origins (Fig. 3c, d). In addition, the distances between the IS and these pre-RC factors recapitulated independent biochemical methods measuring positioning of pre-RC factor binding sites^30–32^, such that the median distances between core IS (peak summit) and ORC1, ORC2 and MCM7 binding sites (peak centre) were 512, 446 and 302 bp, respectively. This positioned the peak of MCM complex downstream of the ORC subunits, at 300 bp from the IS (Fig. 3e). Indeed, the MCM complex sits on at least 68 bp and binds to a neighbouring nucleosome, increasing the size of the protected DNA up to 210 bp^33^. In addition, the MCM helicase must unwind the DNA over a minimum length in order to allow the DNA polymerase to bind to the unwound DNA. We believe that this result, linking the IS determined by SNS-seq and pre-RC binding sites determined by ChIP-seq, is a clear independent demonstration that the SNS-seq method accurately maps the initiation sites of DNA replication. Furthermore, our results show that the relative in vivo positioning of Pre-RC components and IS are similar to those determined by biochemical methods^30^.

### Origin positioning can be predicted based on DNA sequence

As strong origins display a G-rich profile (a putative sequence signature), we asked whether DNA replication origins could be predicted from the DNA sequence alone. Classical motif search algorithms are designed to detect enrichment of short, but highly similar stretches of DNA, typically bound by transcription factors. Given the core origin size (average 716 bp), we hypothesised that they may be specified by hyper-motifs^34^, which are discriminatory DNA sequence patterns that are typically longer than classical transcription factor binding sites. To do this, we modelled the asymmetrical base composition of the core origin and its flanking sequences and scanned the human genome for similar DNA sequence patterns (Supplementary Fig. 3d, see Methods section). The genome scanning (GS) algorithm identified 228,442 non-overlapping regions which located 83% of core origins and 33% of stochastic origins with FPR of 66% (Fig. 3f). The predictive ability of the GS algorithm decreased in parallel with the mean origin activity, suggesting that origins with higher activity (core) are more likely to contain discernible G-rich sequence elements (Fig. 3g). Our GS algorithm also predicted 76% of core and 54% of all origins in the mouse genome (Fig. 3h), which display a similar G-rich sequence signature at core origins (Supplementary Fig. 3e). Asymmetrical base composition at origin sequences has previously been observed^2–4,12^. Interestingly however, only the modelling of core origins, but not of stochastic or previously published origins^2,12^ led to high predictive power with the GS algorithm (see Methods section). In conclusion, despite lack of evolutionary sequence conservation of DNA replication origins in these two mammalian species (Fig. 2k, l), our data suggests that most human and mouse core DNA replication origin positions can be predicted using DNA sequence alone based on the same G-rich DNA hyper-motif, suggesting that a conserved mechanism(s) governs origin selection in these vertebrate species.

To improve the predictive power and reduce FPR, we modelled the DNA sequences around the predicted regions and used two different machine-learning (ML) algorithms (see Methods section) to better differentiate true origins in our predictions. Modelling of the DNA sequences included using information, such as the density of di-, tri- and multi-nucleotides (CC, CG, GG, CGCG, etc.), inter-prediction distances, and the base composition variations (A, T, G, and C) of the DNA across a 4-kb region (see Methods). Remarkably, GS algorithm coupled with a ML algorithm (logistic regression with greedy feature selection, LR) identified 67,297 non-overlapping regions and predicted 67% of core origins with a total FPR 27.8% (Fig. 3i and Supplementary Fig. 3f). In other words, a large proportion (67%) of core origins contain discernible DNA sequence patterns, and when these patterns are present in the genome, they are associated with an origin 72.2% of the time, in at least one cell type. Importantly, when we employed a completely independent ML approach (SVM), this resulted in vastly overlapping predictions (Fig. 3i and Supplementary Fig. 3g) with an FPR of 23.4% (Supplementary Fig. 3f). Coupling of GS and ML algorithms thus allowed the prediction of origin positions in a genome as large as the human genome.

Both SVM and LR approaches identified the upstream G density as critical parameters for predictions (Fig. 3j and Supplementary Fig. 3h). This is in accordance with the presence of an origin G-rich Repeated Element (OGRE)^2^ or tandemly arranged multiple (up to 6–12) G4 structures as well as ultra-short C/G-rich nucleotide motifs found at human, mouse and chicken origins^35^.

### Cell differentiation alters origin positioning and activity

**Fig. 4:**
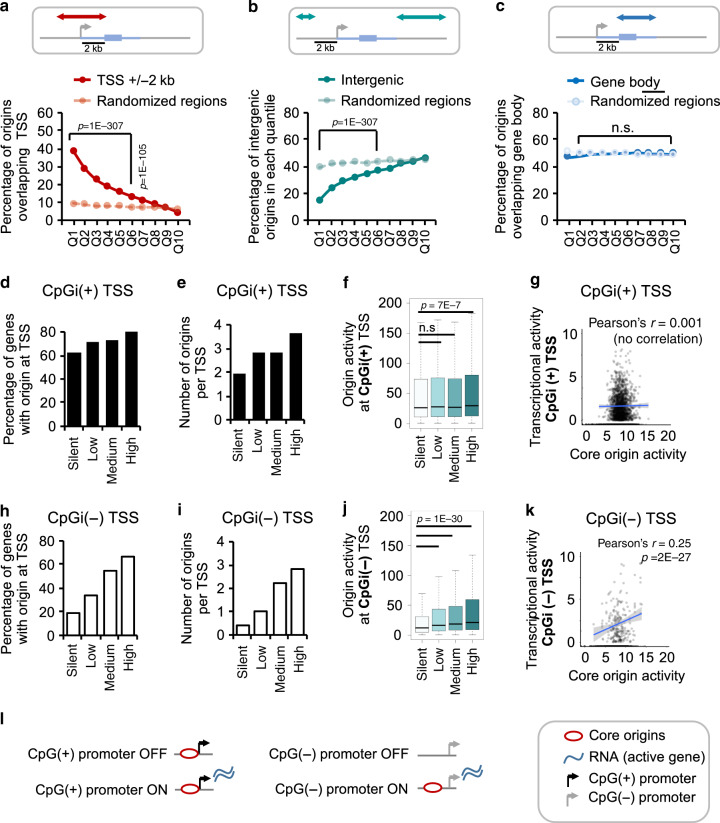
Impact of transcription on the DNA replication origin landscape. **a** Plot representing the percentage of DNA replication origins in each quantile that overlap a promoter region (±2 Kb of TSS) of a GENCODE gene (in red). Overlaps with control regions (paler colour) which are randomly shuffled genomic regions of equal size and number as the origins are also shown. *P*-values obtained by Chi-square Goodness-of-Fit test using observed and expected values for overlap. **b** As in **a** for overlaps with intergenic regions (>2 Kb upstream of a GENCODE gene, TSS are excluded). **c** As in **a** for overlaps with gene body (genic region 2 Kb downstream of the TSS excluded). **d** Bar plot representing percentage of CpG-containing gene promoters that host a DNA replication origin within ±2 Kb of their TSS. Promoters with different transcriptional activity levels in hematopoietic cells are shown (silent = 0, low = 0–15, medium = 15–60, and high = >60 RPKM). In this figure, a promoter is considered CpG-containing (CpG(+)) if a CpG island is present within ±2 Kb of the TSS (Gencode v25). **e** Bar plot showing the average number of origins localised within 2 Kb of the TSS of genes with different transcriptional output levels (silent = 0, low = 0–15, medium = 15–60, and high = >60 RPKM) in hematopoietic cells. **f** Boxplots showing the average activity of origins localised within 2 Kb of the TSS of genes with different transcriptional output levels as in **d** in hematopoietic cells. *P*-values were obtained using the Wilcoxon test in R. **g** Dot plot shows the correlation of transcriptional output of CpGi(+) promoters in hematopoietic progenitors (*y*-axis; RPKMs, Log2) and the activity of core origins located within ±2 Kb of the TSS of these genes in hematopoietic progenitors (*x*-axis; normalised SNS-seq counts, Log2). Top and bottom 5% outliers were removed. The Pearson’s correlation coefficient (*r*) and *P*-value for correlation is indicated on the top, and trendline is shown in blue. **h** As in **d** for CpGi(−) promoter regions. **i** As in **e** for CpGi(−) promoter regions. **j** As in **f** for CpGi(−) promoter regions. **k** As in **g** for CpGi(−) promoter regions. **l** Schematic summary of findings. CpGi(+) promoters (black) tend to host DNA replication origins, irrespectively of their transcriptional status, while CpGi(−) promoters (grey) tend to host origins when they are transcriptionally active.

We observed that in the human genome, core origins were preferentially placed near promoter regions and depleted from intergenic regions (Fig. 4a–c). This is in agreement with a number of studies suggested that transcription is a predictive factor for DNA replication origin specification with varying degrees of correlation^10,24,36–40^. Our data also suggests that in hematopoietic cells, genes with higher transcriptional activity were more likely to host an origin in their promoter region (Supplementary Fig. 4a). Both the number and activity of origins within promoter regions increased with the promoter transcriptional output (Supplementary Fig. 4b, c). Either RNA synthesis activity per se, or open chromatin induced by transcription complex assembly might favour pre-RC formation^14^. However, the correlation between the position of core origins at promoter and intergenic regions (Fig. 4a, b) is not observed for gene bodies (Fig. 4c). This finding suggests an impact of the chromatin environment of the promoter, rather than RNA synthesis per se, in the preferential localisation of origins at promoter regions.

We next used hematopoietic cells undergoing erythropoiesis to examine the impact of changing transcriptional landscape on origin specification. CD34(+) hematopoietic cells were isolated from human cord blood and differentiated towards erythropoietic linage using erythropoietin (EPO; Supplementary Fig. 4d). Gene ontology analysis (GREAT^41^) revealed a single enriched set of genes with origin activity increased upon erythrocyte differentiation (Supplementary Fig. 4e) suggesting that DNA replication origins are recruited to gene domains undergoing transcriptional and epigenetic changes.

### G-rich and transcription impact on origin activity

In HCs, 89% of highly expressed genes hosted a CpGi (a G-rich region) in their promoter, whereas only 48% of silent gene promoters hosted CpGi (Supplementary figure Fig. 4f). Therefore, we asked whether the concomitant presence of a CpGi (or a G-rich stretch) and high transcription activity was required for high origin activity in hematopoietic cells. We did not observe a profound impact of transcription on origin numbers, clustering or activity near CpGi(+) promoters (Fig. 4d–f). In addition, DNA replication initiation activity from CpGi(+) TSS did not correlate with transcriptional activity (Pearson’s *r* < 0.01, Fig. 4g).

In contrast, there is a clear increase in origin positioning at CpGi(−) promoters when the level of transcription is increased (Fig. 4h). Moreover, the number of clustered origins increased proportionally with the transcriptional activity, and the total origin activity was higher with increasing transcriptional activity (Pearson’s correlation *r* = 0.25 Fig. 4i–k). We observed similar trends for gene promoters that contained a G-rich stretch of DNA instead of a CpGi (Supplementary Fig. 4g).

Altogether, these data suggest that the presence of either a CpGi/G-rich stretch or transcription is sufficient to recruit origin activity. In highly active promoters, CpGi or G-rich elements are not correlated with replication origin activity. Conversely, at inactive promoters CpGi/G-rich motifs are clearly associated with replication origin activity (summarised in Fig. 4l). This result is also in line with the presence of G-rich elements at most replication origins.

### Immortalisation results in increased origin positioning stochasticity

**Fig. 5:**
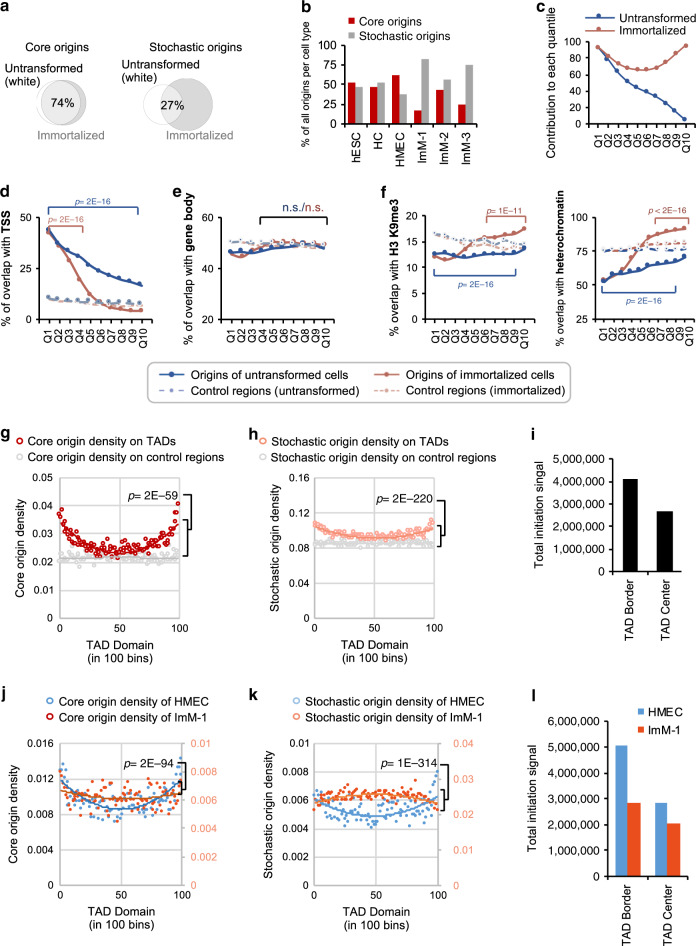
Immortalisation alters the DNA replication origin distribution in heterochromatin and at TAD borders. **a** Euler diagrams showing the percentage of shared core and stochastic origins identified in untransformed (white) and immortalised (grey) cell lines. **b** In immortalised cells stochastic origins are markedly increased. Bar plots showing the percentage of core (red) and stochastic (grey) origins identified in each cell type. **c** Line plot showing the percentage of origins (Q1 to Q10) identified in immortalised (pink) and untransformed (blue) cells. **d** Percentage of origins in each quantile (untransformed Q1–10 in blue, immortalised Q1–Q10 in pink) that overlap with promoter regions (within ±2 kb of the TSS). The expected chance overlap is shown with dotted lines (paler colours). *P*-values obtained by Chi-square Goodness-of-Fit test. *P*-value indicated in blue represent statistical analysis of overlaps in untransformed cells, while pink indicates immortalised cells. **e** As in **d** for overlaps with gene body (excluding the TSS + 2 kb region) of GENCODE (v25) genes. **f** As in **d** for overlaps with regions enriched for heterochromatin-associated H3K9me3 histone mark (in hESC, left panel) and with regions defined as heterochromatin by HMM in hESC and K265 cells (right panel). **g** Plot shows the core origin (red) density across topologically associating domains (TADs)^67^. Average origin density per bin (100 bins) across all TADs was plotted (*y*-axis, in origins/Mb). Core origin density is higher at the TAD borders, creating a “smiley” trend-line. *P*-values were obtained using the non-parametric Wilcoxon test in R. **h** Same as in **g** but for stochastic origins. **i** Bar plot showing the sum of normalised mean SNS-seq signal (*y*-axis, total initiation) across 19 samples coming from both core and stochastic origins at TAD borders and TAD centres. The total amount of SNS-seq signal is 1.53-fold higher at TAD borders. **j** Density of core origins active in HMEC (blue) and ImM-1 cells (orange) across TADs as in **g**. **k** Same as in **j** but for stochastic origins active in HMEC and ImM-1 cells. **l** As in **i** for HMEC (parental, in blue) and immortalised ImM-1 (in orange) cell types.

As aberrant DNA replication is a hallmark of many cancer cells, we next asked whether the origin repertoire was disturbed after cell immortalisation, a key step in cancer development leading to uncontrolled cell proliferation. To this aim, we used three previously described immortalised cell lines obtained by mis-expression of oncogenes^42^ of the parental Human Mammary Epithelial Cell (HMEC) cell line: (i) ImM-1 in which p53 levels was reduced by at least 50% (ΔTP53), (ii) ImM-2 in which the oncogene *RAS* is overexpressed and (iii) ImM-3 in which *WNT* is overexpressed^42^. We identified more origins in the immortalised cell types than in the untransformed cell types (hESC, HC and HMEC; on average 100,000 vs 70,000 origins). This could not be due to higher proliferation rates in these cells as the hESC and HCs proliferated at the same or higher levels (see Methods section). Nevertheless, untransformed and immortalised cell types shared a common core origin repertoire (Fig. 5a) and the bulk of initiation events (~80%) originated from core origins (Supplementary Fig. 5a). The higher number of origins in immortalised cells was clearly caused by an increase in stochastic origins (Fig. 5b). While core (Q1 and Q2) origins were shared between untransformed and immortalised cell types, quantiles with lowest activity (Q8-10) were predominantly contributed by immortalised cell types (Fig. 5c). In order to study origins from untransformed and immortalised cell types disjointedly, we re-classified origins of each category into quantiles separately as described before. Genomic localisation of core origins in relation to genes was comparable in untransformed and immortalised cell lines (Fig. 5d, e). However, stochastic origins from immortalised cells were less enriched near promoter regions (Fig. 5e), but were enriched in heterochromatic regions (marked by K9me3; Fig. 5f). Therefore, immortalisation induces low-activity origins associated with what is heterochromatin in untransformed cells.

Immortalisation also results in differentially up- or down-regulated origins. Strikingly, most down-regulated origins contain G-rich elements such as CpGi/G4, whereas up-regulated origins tend to be G-poor (Supplementary Fig. 5b, c). Therefore, a change in the specification of origins occurs, with preference shifting from G-rich to G-poor DNA for both core and stochastic origins.

We next asked whether there was a specific distribution of core and stochastic origins across topologically associating domains (TADs), which are large regions of the genome that self-interact to form three-dimensional (3D) structures^43^. TAD borders are involved in the insulation of the corresponding chromatin domains, confining chromatin loops inside the TADs, and are enriched in TSS and the insulator factor CTCF^43^. Both human core (Fig. 5g) and stochastic origins (Fig. 5h) were significantly enriched at TAD borders (i.e., “smiley” trend-line). Total amount of DNA replication initiation measured by SNS-seq was also 1.5-fold higher at TAD borders than at TAD centre (Fig. 5i). We obtained similar results for mouse core and stochastic origins (Supplementary Fig. 5d). We conclude that the replication origin density pattern mimics the structural organisation of the genome in individual chromatin domains. This distribution was clearly disturbed in immortalised ImM-1 (TP53^KD^) cells compared with the parental HMEC cell line, and that this variation in origin density on TAD borders was statistically significant (Fig. 5j, k). Total amount of replication initiation at TAD borders and TAD centre was also markedly different in the ImM-1 cells compared to the parental HMEC (Fig. 5l). hES cells, or other untransformed cell types did not display altered core origin density at TAD borders, suggesting that this property is specific to immortalisation and does not reflect high proliferation rates (Supplementary Fig. 5e).

## Discussion

DNA replication origin specification remains poorly understood despite the progress in next-generation sequencing technology that allowed IS mapping genome-wide. In this study, we used the SNS-Seq method, which has the highest resolution to map replication origins^1^, in which the signal was corrected with suitable experimental controls generated in parallel (see Methods section). We found a remarkable consistency in the specification of a subset of IS, termed core origins, in multiple cell types that is maintained even after immortalisation. Core origins, which represent ~30,000 regions in any given cell type, hosted the bulk of DNA replication initiation events (70–85%) in all the studied cell types. We uncovered that most core origins could be predicted by a computational algorithm based only on sequence recognition, thus unequivocally concluding that replication origins are preferentially activated in a precise set of regions in mammalian genomes in different cell types.

Our study also reveals that the underlying DNA sequence is a prominent predictor of origin positioning in the human and mouse genomes. The G-rich sequence patterns commonly found in core origins were predictive of origin placement genome-wide. When present in the human genome, 72% of these patterns were associated with DNA replication initiation in at least one cell type. The stretch of G-rich repeated DNA sequence (OGRE) upstream of the IS corresponds with ORC1, ORC2 and MCM2-7 binding regions, coupled to a region with lower G and C content (Fig. 3b–e). Core origins are also often clustered, suggesting that they represent regions of the genome with several potential pre-RC binding sites. This organisation might constitute a broader pre-RC binding platform that may host several pre-RC and increase the efficiency of MCM loading and origin activation. Conversely, most stochastic origins contain a shorter stretch of G-rich region, possibly representing single putative pre-RC binding sites (Fig. 3b). The position of the initiation sites revealed by SNS-seq is in perfect agreement with the positions of pre-RC factors determined independently^30–32^, which are found upstream of the initiation site, coinciding with the G-rich region as expected, (Fig. 3e). Importantly, this finding is an independent confirmation of the association of G-rich regions to metazoan replication origins.

How can a G-rich region be involved in initiation of DNA replication? One formal possibility for G-rich SNS-seq peaks could be the experimental protocol involving the use of lambda exonuclease, where G-rich sequences could be resistant to digestion^44^. However, the experimental conditions for SNS-seq used in most studies, including ours but excluding the aforementioned study, are stringent (see Methods section). Moreover, control SNS-seq samples treated in parallel (+RNase) are only slightly enriched in G-rich DNA. In addition, the G-rich nature of replication origins has been also confirmed using a nascent strand purification method that does not employ lambda exonuclease^7^. Finally, some factors involved in initiation of DNA replication co-localise with DNA replication origins (this study) and can bind to G4 (see below).

A second possibility may be linked to the ON/OFF stages of DNA replication origins. The opening of DNA at the replication initiation sites requires two temporally successive steps^45,46^. First, Pre-RCs form in G1, through the binding of ORC, Cdc6 and Cdt1, which permit the recruitment of the MCM helicase. It is accepted that all potential origins are pre-set at this stage, but it is still not known how the metazoan origins are recognised by the ORC. The activation of the MCM helicase occurs at the G1-S transition, but only 20–30% of the pre-RCs are activated in S phase. A fundamental characteristic of G4 is its ability to form several structures^47^, including folded and unfolded forms. These two forms might regulate the OFF stage (pre-RC) or the ON stage (initiation) of a replication origin; Exogenous G4 sequences able to form G4 structures do not inhibit the formation of pre-RCs in Xenopus egg extracts, but do compete with the firing of replication origins^48^. This result may suggest that the folded form of G4 participates in the initiation of DNA synthesis but is not required for origin recognition by pre-RC proteins. In agreement, MTBP, RecqL and Rif1, three factors involved in origin firing, all bind to G4^49–53^.

A third possibility is guided by the NS profile at replication origins which may suggest that G4 act as a transient pause of the replication fork initiating at replication origins. Several previous studies have reported the enrichment of G-rich regions 5′ to the initiation site^2,3,5,54^ and suggested a transient pause of the replication fork at the G4. This hypothesis suggests that the G-rich/G4 structures are folded when origins are activated and then unfolded through a mechanism imposing a transient pause of the progressing replication fork, a phenomenon similar to transcriptional pausing^55^. Whether such a mechanism exists or has a regulatory function as a checkpoint on the progression of the replication machinery remains to be demonstrated.

The finding that the underlying DNA sequence is predictive of origin placement in a given species naturally leads to question to which extent chromatin and transcriptional environment is also involved in initiation of DNA replication. Origin positioning has previously been correlated with open chromatin and various histone marks related to active chromatin^2^. Core origins often coincide with transcription and regulatory elements of the genome (e.g., promoters and enhancers; Fig. 4a and Supplementary Figure Fig. 5f) that are associated with activating histone marks and open chromatin^56^. It is conceivable that the DNA sequence pattern we identified is usually part of open or permissive chromatin. However, core origins are also present in non-genic regions (19.4%) or silent genes. In addition, the impact of transcription and the presence of a G-rich element can be uncoupled. The presence of a G-rich element/CpGi in the promoter region of silent genes, or in non-coding regions, is sufficient to host replication origin activity. Of note, polycomb group proteins associate with CpGi(+) promoters and can bind to G4 DNA^57^. We previously showed that the presence of these proteins is a strong indicator of origin positioning, supporting a mechanism by which silent CpGi(+) gene promoters or repressed chromatin may host origins. Interestingly a recent report also supports a role for G4 elements in the regulation of polycomb-mediated gene repression^58^. In conclusion, even though the DNA sequence information is not as strictly defined as the consensus ARS element sequence present at *S*. *Cerevisiae* origins^1^, its predictive value shows that sequence specificity is a conserved feature of replication origins in metazoan cells. We also acknowledge that a combination of select epigenetic marks together with sequence information might improve the prediction of metazoan replication origins.

Besides core origins, which represent most of the SNS signal, our analysis also identified thousands of stochastic origins, which poorly coincide with G-rich elements. Interestingly, immortalisation greatly increased the number of these low-activity origins, especially within heterochromatic regions. This was accompanied by equalisation of DNA replication initiation events at TAD borders and centres (Fig. 5l).

The finding that replication origins are enriched at TAD borders might reflect a role for DNA replication origins in the formation of chromatin loops or their consequence. As such, density of origins could play a role in the insulation of replication domains. This is also reminiscent of previous findings that origin density/origin activity is highly correlated with replication timing^3,25^. In addition, replication timing boundaries correlate with TAD boundaries^49^. Hence, altered DNA initiation density, aberrant replication timing and altered chromosomal structure organisation might be linked in cell types undergoing immortalisation. A previous study linked mis-expression of the oncogenes *MYC* and *CCNE1* to formation of intragenic origins upon premature S-phase entry in a tumour-derived cell line^59^. Here, we show that both the number and distribution of replication origins is perturbed during immortalisation, an important step in cellular transformation. Both the increased stochasticity in origin placement and perturbation of the DNA replication initiation density profile on TADs could therefore be new landmarks associated to cancer cells.

## Methods

### Cell and tissue culture

H9 hESC cells (WA-09; Wicell) were obtained from ES Cell International (ESI, Singapore) and were maintained according to supplier’s instructions, as described^60^. Briefly, undifferentiated hESC were grown on mitomycin C-treated (10 g/ml, Sigma) mouse embryonic fibroblasts (used at the cell density of 4–6 × 10^4^ cells/cm^2^) and in medium constituted by 80% Knock-Out DMEM, 20% Knock-Out Serum Replacement, 1% non-essential amino acids, 1mM l-glutamine and 0.1 mM β-mercaptoethanol. At passaging, 8 ng/ml human bFGF (Millipore or Eurobio) was added to the medium. Peripheral blood mononuclear cells (referred to as hematopoietic cells, HC) were isolated from the umbilical cord blood of three independent human donors from the Clinique Saint Roch of Montpellier using the Ficoll density gradient method^61^. HC were then purified by magnetic beads coupled with an anti-CD34 antibody, resulting in 0.5 to 1 × 10^6^ CD34+ cells, plated in culture and expanded ex vivo with supplemented Stem Span medium (IMDM + insulin, transferrin, BSA, 5% FCS + IL-3 + IL6 + SCF) for 6–7 days. Cell differentiation towards the erythropoietic lineage was induced by addition of erythropoietin (EPO, 3 units/mL). At different time points after EPO addition (day 0, 3 and 6), an aliquot of 50 × 10^6^ cells was collected and pelleted for molecular biology experiments (SNS-Seq, RNA-seq and RT-qPCRs for verification), while the remaining cells were left in culture. To verify erythropoietic differentiation, cells were phenotyped by flow cytometry analysis using antibodies against the hematopoietic/erythroid markers CD36, CD11b, GlyA, CD71, CD49d, CD34, CD98, IL3R and CD13 (Beckman Coulter). Differentiation into the erythrocyte linage upon EPO incubation was also confirmed by RT-qPCR analysis of RNA from cells at day 0, 3 and 6 using primers specific for linage markers. HMEC cells were isolated and ImM1-3 cells were generated as previously described (available at https://www.biorxiv.org/content/early/2018/06/11/344465). Briefly, HMEC cells were initially immortalised using a stably transfected shRNA against *TP53* (ImM-1). ImM-1 subclones were then generated by stable transfection of plasmids to over-express human *RAS* (ImM-2) or *WNT* (ImM-3).

Mouse ESC were cultured as previously described, and SNS-seq was carried^2^ on mESC (*n* = 4) and neuronal progenitor cells (*n* = 4). A total of 248,682 origins were identified and divided into 10 equal size quantiles as in human.

### Ethical permissions

All experiments, including those involving hESC and hematopoietic cells adhere to the guidelines established by the French Bioethics Laws, and the “Agence Française de biomedicine”. CD34+ cells were isolated from umbilical cord blood obtained following delivery of de-identified full-term infants after written informed consent from the mothers. Use of these de-identified samples was determined to be exempt from ethical review by the University Hospital of Montpellier Institutional Review Board in accordance with the guidelines issued by the Office of Human Research Protections.

### Nascent strand isolation (SNS-seq) and analysis

This method is the most precise procedure to map replication origins, although differences in SNS-seq and bioinformatics analysis methodologies, often using no or unsuitable controls, have affected the false-positive rate (FPR) in origin identification, resulting in varying properties attributed to metazoan origins^4,10,13,15–17,44^. Here, we are providing our SNS-seq protocol and an analysis pipeline. Briefly, cells were lysed with DNAzol, and then nascent strands were separated from genomic DNA based on sucrose gradient size fractionation^2^. Fractions corresponding to 0.5–2 kb were pooled, incubated with T4 polynucleotide kinase (NEB) for 5′ end phosphorylation, and digested by overnight incubation with 140 units of λ-exonuclease (λ^exn^). A second round of overnight digestion with 100 units of λ^exn^ was performed. λ^exn^ digests contaminating broken genomic DNA, but not RNA-primed nascent strands^22^. As experimental background control, high molecular weight genomic DNA for each cell type was heat-fragmented to the same size as nascent strands, incubated with RNase A/XRN-1 to remove the RNA primer in any contaminating nascent strand, and then treated with the same amounts of λ^exn^ as the samples.

We should stress that the conditions ours and most laboratories use for the SNS-Seq are strictly different from the report claiming a possible bias of the lambda exonuclease digestion^44^. First, in classical SNS-Seq protocols, nascent RNA-primed at replication origins are purified by melting DNA followed by the separation of the nascent strands from the bulk parental DNA by sucrose gradient centrifugation. Only then, the purified nascent strands are digested with exhaustive lambda exonuclease digestion (more than 2000 u/μg DNA). This is not the case in Foulk et al.^44^ in which bulk DNA is simply enriched in replication intermediates by using BND cellulose, which fractionates whole DNA that is partly single stranded. Lambda exonuclease is then used, resulting in an enzyme to DNA ratio 1000–3000-fold less than the ratio our laboratory employs. We also repeatedly reported that all our control samples (Nascent strands from mitotic DNA, or G0 DNA, or high molecular weight DNA give very low enrichment values^2,4,22,48,62^).

The quality of origin enrichment in each sample was first tested by qPCR using primers against known human replication origins. Primers used to detect origin activity for various origins are given in Supplementary Data 4. Single stranded nascent strands were first purified using the CyScrib GFX Purification Kit (Illustra, 279606-02), then converted into double-stranded DNA by random priming using DNA polymerase I (Klenow fragment) and the ArrayCGH Kit (Bioprime, 45–0048). cDNA libraries were prepared using the TrueSeq Chip Library Preparation Kit (Illumina). In parallel, heat-denatured genomic DNA input controls were also purified, random-primed and libraries prepared in the same manner. All samples were sequenced at the Montpellier GenomiX (MGX) facility using an Illumina HiSeq 2500 apparatus. bcl2fastq version 2.17 from Illumina was used to produce the fastq files. Illumina reads (50 bp, single-end) from each SNS-seq replicate were trimmed and aligned to hg38 using Bowtie^2^ (v2.2.6). Peaks were called using two peak calling programs: MACS2^63^ (v2.2.1) and SICER^64^ (v1.1 modified to contain hg38 and mm10). Peaks were first called using MACS2 (default parameters plus–bw 500 -p 1e-5 -s 60 -m 10 30–gsize 2.7e9), followed by peak calling by SICER [parameters: redundancy threshold = 1, window size (bp) = 200, fragment size = 150, effective genome fraction = 0.85, gap size (bp) = 600 and FDR = 1e-3]. MACS2 peaks that intersect SICER peaks from each sample were merged using bedtools intersect to generate a comprehensive list of all human DNA initiation sites (IS; Table 1). Blacklisted regions as defined by the ENCODE project (hg38, ENCSR636HFF) were subtracted from the final human DNA replication origin list. Mouse SNS-seq samples were processed as human SNS-seq and were also divided into quantiles (mQ1-mQ10) with each quantile containing 25,168 regions. Principal component and analysis and sample distances suggest that for cell types obtained from a single donor (i.e. HMEC), the overlap of origins is stronger amongst the replicates, than it is with other cell types. For donor-derived cell type (hematopoietic cells), we observed that the SNS-seq samples are more similar within the same donor than with treatment status (i.e. treatment with EPO). This is in contrast with the RNA-seq data, where samples cluster according to their treatment (EPO) and not their origin (donor).

### SNS-seq optimisation and quality controls

Different experimental and bioinformatics methodologies have been used to obtain and analyse SNS-seq data. SNS-seq relies on the λ^exn^ ability to specifically digest genomic DNA, while leaving the newly synthesised, RNA-primed nascent DNA intact. Our analysis suggests that peak calling to define origin locations using 19 human SNS-seq samples in the absence of a background or experimental genomic DNA background identified ~200,000 and 150,000 peaks per sample respectively (mean number of peaks). This number is reduced by about half when an appropriate experimental background (heat-fragmented genomic DNA treated with RNAse and λ^exn^) is used, suggesting that the use of appropriate backgrounds is crucial to reduce false positives in peak-calling. When we examined the nature of the background signal (RNAse + λ^exn^), we observed only a minimal bias for G-rich regions (G4, G-rich and CG-rich) compared with randomised genomic regions (~5 reads every 250 bp compared to ~2 reads per 250 bp), a value insufficient to skew peak calling or the downstream analysis. This confirms that under our experimental conditions (in particular our λ^exn^ digestion conditions), putative G4, G-rich and GC-rich sequences are digested almost as efficiently as randomised DNA sequences, and that the background generated by regions resistant to digestion can be accounted for by using a suitable experimental background sample.

### Summits and orientation of origins

Summits of origins were defined by calculating the highest number of SNS-seq reads in bins of 50 bp from 25 bp sliding windows, using bam files from all samples with a custom-made script (see code availability). Middle point of the bin with highest number of reads was considered the summit of the IS. Origins were assigned a plus or a minus strand based on the G-content of the regions flanking the IS summit, such that the G-rich flanking region was oriented upstream (left) of the IS summit. To do this, we calculated the number of G bases within 500 bp of each IS and assigned a (+) or a (−) strand to each origin to ensure that the 500 bp with the most number of G bases was oriented upstream of the IS.

### Quantification, classification, and differential activity of DNA replication origins

The bioinformatics on this project was supported by the high power computing cluster of University of Birmingham (CastLes and BlueBear). Quantification of the SNS-seq signal at DNA replication origins was done using the R-package DiffBind (v3.9, dba.sCore: TMM_minus_background), using all human/mouse origin coordinates. The TMM_minus command subtracted the background signal from the signal, before normalising all 19 samples using a TMM based algorithm. “Normalised SNS-seq signal” in the manuscript refers to these values obtained after subtraction of background and TMM normalization. After the TMM normalisation, the average normalised SNS-seq counts was calculated across the 19 samples for each origin and origins were ranked based on this value. Then, each origin was assigned to a quantile (Q1–Q10) that represents the origin position in the ranked list based on the average activity. For example, all origins in the top 10th percentile of activity were assigned to Q1, and all origins that ranked between the 10th and 20th percentile were in Q2, and so forth. Core origins were all Q1 and Q2 origins, while stochastic origins were in all the other quantiles (Q3–Q10). Super origins were defined as having >50 normalised SNS-seq counts. Super origins were not included in the present analysis, but they are listed in Supplementary Data 1, for readers interested in origins that are ultra-ubiquitous in the genome, such as the MYC and LaminB2 origins.

To determine the percentage of SNS-seq signal that falls in Core origins in each cell type, the total normalised (background subtracted and normalised) SNS-seq signal and the fraction that belongs to Q1, Q2 and stochastic origins (Q3–Q10) were calculated.

Differential origin activity was calculated using the R libraries Diffbind (v3.9, TMM_minus) and DeSeq2 consecutively (see code availability for code).

### Total initiation from early and late replicating domains

The early and late replicating domains were defined based on early and late replication domains common to H9 and CD34+ hematopoietic progenitors (Supplementary Data 3). The origin coordinates (±2 kb) were removed (masked) from the domains. The SNS-seq signal was then quantified in these domains in both sample and background samples and normalised by RPKM. The signal was then calculated as: Total SNS-seq signal in sample over early replicating domains minus the Total SNS-seq signal in background over early replicating domains. The same was performed for late replicating domains. The average of three replicates was calculated for each cell type. For most cell types, the signal from non-origin replication domains did not exceed the background (i.e. was negative).

For hESC and IMM-1, where we find that the initiation signal from early or late (respectively) replication domains exceeds the background, we calculated the percentage of initiation from non-origin regions and origin regions and presented it in Supplementary Fig. 1d.

### Clustering of core origins

Clustering of core origins was done using bedtools suite (v.2.25, command:bedtools cluster) with a maximal distance of 7 kb to the nearest core origin. Please note that bedtools does not perform categorical clustering. Supplementary Fig. 2e shows a diagram for clustering. This means that 70% of core origins were found in clusters with at least two or more core origins that are at a maximal distance of 7 kb from another core origin. Isolated core origins, which make up 15% of core origins, are found more than 15 kb away from another core origin. We also defined “loosely clustered” core origins, which were <15 kb but >7 kb to nearest core origin. Comparison with OK-seq data: in order to define tightly clustered core origins, we screened core origin clusters for those that contained six or more core origins. This produced 1039 clusters with an average size of 27,287 bp that contained 13,519 core origins. As OK-seq did not map X- and Y-chromosomes, we also removed clusters mapping to these chromosomes for this comparison. The size of tight core origin clusters is comparable to the average initiation zone defined by OK-seq, which is ~34 kb in size.

### Distance between IS and Pre-RC components

Peak coordinates were downloaded from relevant sources (ORC1^24^, ORC2^25^ and MCM7^26^) and mapped to hg38 version of the human genome. For ORC2 peaks, we were provided with peak summits, while for ORC1 and MCM7 peaks peak centre was calculated as the peak summit. For overlaps with ORC1 and ORC2, peaks were extended ± 2 kb. In order to map the density of distance between Pre-RC components and IS summit, we calculated the distance between the IS summit and the ORC2 summit or ORC1/MCM7 peak centre for all Pre-RC components within a distance of 10 kb of the IS. We then plotted the density of these distances in R. As a control, this procedure was repeated with randomised genomic coordinates for pre-RC components, which did not show any enrichment upstream or downstream of IS.

### Data analysis and plotting

Heatmaps, boxplots, and other plots were generated using ggplot2 (v3.1.0) and pheatmap (v1.0.12) in R. Pie charts were generated in Excel (v16.16.23) using data obtained in R. Both Pearson’s and Spearman’s correlation matrices were calculated in R using (command cor()). Principal component analysis (PCA) and Euler diagrams were generated in R (command pca, library eulerr). Comparison between genomic coordinates (quantiles, alternative origin mapping methods and histone/Pre-RC binding sites; intersectBed with a minimum overlap of 1 bp) as well as generation of randomised genomic coordinates were computed using the bedtools suite (bedtools shuffle –chrom, -noOverlapping, when possible). For computation of overlaps between ORC1 and ORC2 binding sites and origins, a maximum distance of 2 kb was taken as positive overlap. SNS-seq read density plots and heatmaps were generated using deeptools (plotProfile, plotHeatmap). When required, genome coordinates of different genome assemblies were converted using UCSC LiftOver (UCSC Toolkit). A full list of the genomic regions downloaded from external sources can be found in Supplementary Data 3.

### ReMap and putative enhancers

Origins were mapped onto the ReMap atlas^56^ (http://remap.cisreg.eu). ReMap results from an integrative analysis of transcriptional regulator ChIP-seq experiments from both Public and Encode datasets. The ReMap catalogue includes 80 million peaks from 485 transcription factors, transcription coactivators and chromatin-remodelling factors. Overlaps were assessed with bedtools (v.2.25), counting only regions with a minimum of 10 ChIP-seq peak overlap.

### RNA-seq and analysis

RNA-seq profiling was performed on all HC samples in order to determine whether origin positions (SNS-Seq) are adapted with transcription programs (RNA-seq). To do so, ≥2 µg RNA was extracted and purified from an aliquot of 200,000 cells using TRIzol reagent (Sigma-Aldrich), followed by RNA purification using the RNEasy MiniKit (Qiagen 74104). RNA quality and quantity were analysed using a Fragment Analyzer (Advanced Analytical). cDNA libraries were prepared by the Montpellier GenomiX facility using the TrueSeq Chip Library Preparation Kit (Illumina). After quality control (using FastQC v0.11.5), the TopHat software (version 2.1.1) was used for splice junction mapping through Bowtie2 (version 2.2.8) for mapping reads. Reads count on genes was performed using HTSeq-count (version 0.6.1p1). Gene annotations were downloaded from GENCODE, release 25 (GRCh38.p7, 23 September 2016). Data were normalised by the relative log expression implemented in edgeR (version 3.8.6), and pairwise comparative statistical analysis to identify differential genes was performed using DeSeq2 (version 1.18.0 in R 3.2; results were confirmed with edgeR version 3.8.6) using a generalised linear model.

### Definition of G-rich regions (G4, CpGi, G-rich)

Two methods were used to define G4 elements in the human genome based on (i) identification of mismatches induced by K^+^ and pyridostatin (PDS) treatment^28^ (in vitro G4) (ii) predictions by G4Hunter^29^ (in silico G4). Both datasets were generated in hg19, therefore we have converted our origin coordinates to hg19 in order to examine overlaps. CpG islands that were >300 bp in size were downloaded from UCSC (hg38). G-rich regions were defined as having a G density >37% within a 500-bp window in sliding windows of 100 bp (hg38) using bedtools commands bedtools makewindows, nuc and count. G-rich region list was used for the analysis in Supplementary Fig. 4d.

### Analysis of base composition and motif discovery in genomic regions

Base composition was analysed using HOMER^65^, with 100 bp as window size taking the IS summit as the peak centre. The density data were visualised with Microsoft Excel. HOMER (v4.11.1) was used to search for motif enrichment in between the core origin summits and the 400 bp upstream regions (in oriented origins, this corresponds to the G-rich region). We have used the following parameters; perl findMotifsGenome.pl hg38 -size given -len 4,6,8,10,12 -mask -norevopp [none, -noweight or –CpG].

### Evolutionary conservation analysis

Refseq exons, introns and promoter regions (defined as −500 to 0 bp upstream of transcription start sites) and Phastcon scores (Phastcon20way) were downloaded from UCSC table browser (last update 12/2017). Mean cumulative phastcon scores of each set of regions were calculated using R and bedtools suite (bedtools coverage). Human origin coordinates were converted to mouse coordinates either using LiftOver (UCSC toolkit) or BLAST. Very similar results were obtained with BLAST and LiftOver, we presented the results from LiftOver.

### Prediction of DNA replication origins in the human and mouse genomes

The human and mouse genomes were divided into paired 500 bp windows (Watson and Crick strands separately) with a sliding window size of 100 bp using bedtools (makewindows) suite (~30 Million windows for human genome). The number of each nucleotide (A,C,G,T) in each paired window was then calculated (bedtools nuc). Paired (consecutive) 500 bp windows were evaluated to fit a DNA sequence pattern (a hyper-motif) with minimum 28% G in the first window and minimum 25% G in the consecutive second window – and a requirement that G content drop by 8–40%, with a max A/T content 0.21 between the first and second window). This let us to identify 1,041,594 window pairs. The window pairs that were retained were then merged using bedtools merge to identify non-overlapping putative origin regions (228,442 regions with average size of 1.7 Kb).

### Prediction of DNA replication origins in the human and mouse genomes Genome Scan Algorithm

The human and mouse genomes were divided into paired 500 bp windows (Watson and Crick strands separately) with a sliding window size of 100 bp using bedtools (makewindows) suite (~30 Million windows for human genome and hg38). The number of each nucleotide (A,C,G,T) in each paired window was then calculated (bedtools nuc). Paired (consecutive) 500 bp windows were evaluated to fit a DNA sequence pattern (a hyper-motif) with minimum 28% G in the first window and minimum 25% G in the consecutive second window – and a requirement that G content drop by 8–40%, with a max A/T content 0.21 between the first and second window). The same algorithm was run for the reverse compliment strand (i.e. Crick strand, 28% C in second window, min 25% C in second window) on the same 30 M window pairs, bringing the number of window pairs examined to 60 million.

This let us to identify 1,041,594 window pairs. The window pairs that were retained were then merged using “bedtools merge” to identify non-overlapping putative origin regions (228,442 regions with average size of 1.7 Kb). This set of regions was used to define predictability of origins in Fig. 3f, g. For the mouse genome, the same algorithm was run with exactly the same parameters, which retains 689,285 window pairs out of the (27 × 2 million possible pairs from mm10). Similarly, these regions were merged (bedtools merge) to generate 230,052 non-overlapping regions and intersected with mouse origins using bedtools (bedtools intersect –wa –u) to generate Fig. 3h.

### Machine learning and hyper-motif analysis

Predicted variable for our algorithm is the membership to the “origins” class defined by intersection of the non-overlapping coordinates with an origin (maximising the predictive power on core origins in particular). In all, 30 million pairs of 500 bp windows were randomly split into two equally sized datasets. One of the datasets was reserved for the final validation at the end of the model development (test set). The other set was used for training and internal validation of the prediction model. Next, the training set was randomly split into 10 non-intersecting subsets and 10-fold internal cross-validation was performed (i.e. used nine of these subsets for internal training and the remaining one for internal validation of the models, this was repeated 10 times, each time with a different validation subset). Initially, the Genome Scan algorithm was run on each one of those 10 internal training datasets. On the set of 1,041,594 regions generated by the GS algorithm (window pairs, see above), we constructed a set of 22 parameters/predictors (see Supplementary Data 2) using domain knowledge. Then, machine learning procedures were applied to the output of the Genome Scan, thereby constructing a hierarchical classifier. This procedure was repeated 100 times for two different machine-learning algorithms (i) logistic regression with greedy incremental feature and (ii) support vector machines with lasso regularisation. Greedy feature selection was performed by means of a modified version of statistical R-package CARRoT (Predicting Categorical and Continuous Outcomes Using One in Ten Rule, R CRAN package, 2018, Alina Bazarova and Marko Raseta, v1.0). The software was modified in such a way that would allow to incorporate merging of the output into non-intersecting genome regions by means of bedtools and then assessing the predictive power of the model given these regions. The support vector machine prediction was performed using R-package sparseSVM^66^ and additional scripting described above.

We chose the models aiming at maximising their balanced (average class-wise) accuracy defined as 0.5*[TP/(TP + FN) + TN/(TN + FP)], where TP, TN, FP and FN stand for True Positives, True Negatives, False Positives and False Negatives. Due to the absence of the synthetically constructed negative instances of the origins these quantities were computed in terms of the overall length of the regions corresponding to true positive, true negative, false-positive and false negative hits of 500 bp window pairs. We kept on adding features to the greedy feature selection until improvement in predictive power was lower than 10^−3^. When working with SVM we chose penalising parameters which led to the highest cross-validated predictive power as defined above. At the end of the procedure we obtained 100 predictive models for each method which exhibited the highest predictive power for a given 10-fold cross-validation partition. For logistic regression, the best model emerged with the highest frequency of the predictors constituted by the features: UP_C_fraction, UP_G_fraction, Down_T_fraction, G_content_2kb, rampG, AAA, GG and TTT (Supplementary Data 2). Once the training was complete, the chosen models based on 10-fold cross-validation were fitted with the whole original training set of 15 million pairs of 500 bp windows. The resulting trained models were then tested on the final hold-out test set (isolated from the training one in the very beginning and never touched throughout the model construction phase). Please note that each algorithm reported non-duplicate window pairs (i.e. if a window pair is retained with both forward and reverse scanning procedure by the genome scan algorithm, this window pair is reported once as positive by either machine-learning algorithm).

In order to generate the predictions genome-wide, the trained model was run on the entire set of regions from GS resulting in 333,986 window pairs for LR and 279,195 window pairs for SVM called as positives by each algorithm. These window pairs were merged using bedtools (bedtools merge) to generate non-overlapping windows of 67,297 (LR) and 57,339 (SVM) regions. Please note that due to the sliding window pattern we used to scan the genome, each window overlays 9 other windows, thus the same genomic regions are reported numerous times. We remove the repeating regions by merging them, using bedtools merge, thus obtaining non-overlapping regions of the genome. These non-overlapping regions were used to generate the final predicted regions (i.e. Fig. 3i for core origins) or total false-positive rate (regions not intersecting an origin, Supplementary Fig. 3f, normalised to average fragment length).

### Calculation of origin density and total initiation signal across TAD domains

To calculate the origin density across TAD domains, each TAD was divided into 100 bins (bedtools makewindows –n 100). As the bin size in each TAD was a fraction of the TAD size, the number of origins in each bin of the TAD was normalised to the bin size. To determine whether origin density across the TAD was significantly different in different cell types, the origin density across TADs for each bin was normalised to the 20 bins in the middle of each TAD (bin numbers 40–60). These values represent the differential origin density between the TAD middle and borders, rather than the overall origin density across the TAD. We have calculated the sum of normalised (background subtracted) signal from origin regions that fall onto TAD borders or TAD centres (dataset on Table 3 and Fig. 5i, l). As before, TAD domains were divided into 100 bins and the 20 bins (1–10, 91–100) were defined as borders, while 20 bins (41–60) were considered as centres.

### Statistical significance

Different statistical tests were used depending on the data nature, as indicated in the figure legends. Specifically, the R commands “wilcoxon.test”, “t.test” and “chisq.test” were used to measure statistical significance. *p* = 1E-307 and *p* = 2E-16 represent the lowest value stored in the memory of R (depending on the version). The Chi.square test is essentially a one-sided test, while Wilcoxon assumes a non-parametric distribution.

### Reporting summary

Further information on research design is available in the Nature Research Reporting Summary linked to this article.

## Supplementary information

Supplementary Information

Description of Additional Supplementary Files

Supplementary Data 1

Supplementary Data 2

Supplementary Data 3

Supplementary Data 4

Supplementary Data 5

Reporting Summary

## Data Availability

Data downloaded from external sources can be found in Supplementary Data 3. Raw read files for SNS-seq/RNA-seq and processed files can be found at the NCBI Gene Expression Omnibus (GEO) under the accession code GSE128477. All data is available from the authors upon reasonable request.

## Code availability

Scripts and other bioinformatics pipelines used to analyse SNS-seq data can be found at https://github.com/iakerman/SNS-seq or available from the authors upon reasonable request.

## References

[1] Ganier O, Prorok P, Akerman I, Mechali M (2019). Metazoan DNA replication origins. Curr. Opin. Cell Biol..

[2] Cayrou C (2015). The chromatin environment shapes DNA replication origin organization and defines origin classes. Genome Res..

[3] Cayrou C (2012). New insights into replication origin characteristics in metazoans. Cell Cycle.

[4] Cayrou C (2011). Genome-scale analysis of metazoan replication origins reveals their organization in specific but flexible sites defined by conserved features. Genome Res..

[5] Comoglio F (2015). High-resolution profiling of Drosophila replication start sites reveals a DNA shape and chromatin signature of metazoan origins. Cell Rep..

[6] Krasinska L (2008). Cdk1 and Cdk2 activity levels determine the efficiency of replication origin firing in Xenopus. EMBO J..

[7] Langley AR, Graf S, Smith JC, Krude T (2016). Genome-wide identification and characterisation of human DNA replication origins by initiation site sequencing (ini-seq). Nucleic Acids Res..

[8] Tubbs A (2018). Dual roles of poly(dA:dT) tracts in replication initiation and fork collapse. Cell.

[9] Delgado S, Gomez M, Bird A, Antequera F (1998). Initiation of DNA replication at CpG islands in mammalian chromosomes. EMBO J..

[10] Sequeira-Mendes J (2009). Transcription initiation activity sets replication origin efficiency in mammalian cells. PLoS Genet..

[11] Costas C (2011). Genome-wide mapping of Arabidopsis thaliana origins of DNA replication and their associated epigenetic marks. Nat. Struct. Mol. Biol..

[12] Picard F (2014). The spatiotemporal program of DNA replication is associated with specific combinations of chromatin marks in human cells. PLoS Genet..

[13] Besnard E (2012). Unraveling cell type-specific and reprogrammable human replication origin signatures associated with G-quadruplex consensus motifs. Nat. Struct. Mol. Biol..

[14] Mechali M (2010). Eukaryotic DNA replication origins: many choices for appropriate answers. Nat. Rev. Mol. Cell Biol..

[15] Martin MM (2011). Genome-wide depletion of replication initiation events in highly transcribed regions. Genome Res..

[16] Gomez M, Brockdorff N (2004). Heterochromatin on the inactive X chromosome delays replication timing without affecting origin usage. Proc. Natl Acad. Sci. USA.

[17] Smith OK (2016). Distinct epigenetic features of differentiation-regulated replication origins. Epigenet. Chromatin.

[18] Giacca M (1994). Fine mapping of a replication origin of human DNA. Proc. Natl Acad. Sci. USA.

[19] Vassilev L, Johnson EM (1990). An initiation zone of chromosomal DNA replication located upstream of the c-myc gene in proliferating HeLa cells. Mol. Cell Biol..

[20] Ladenburger EM, Keller C, Knippers R (2002). Identification of a binding region for human origin recognition complex proteins 1 and 2 that coincides with an origin of DNA replication. Mol. Cell Biol..

[21] Taira T, Iguchi-Ariga SM, Ariga H (1994). A novel DNA replication origin identified in the human heat shock protein 70 gene promoter. Mol. Cell Biol..

[22] Cayrou C, Gregoire D, Coulombe P, Danis E, Mechali M (2012). Genome-scale identification of active DNA replication origins. Methods.

[23] Petryk N (2016). Replication landscape of the human genome. Nat. Commun..

[24] Dellino GI (2013). Genome-wide mapping of human DNA-replication origins: levels of transcription at ORC1 sites regulate origin selection and replication timing. Genome Res..

[25] Miotto B, Ji Z, Struhl K (2016). Selectivity of ORC binding sites and the relation to replication timing, fragile sites, and deletions in cancers. Proc. Natl Acad. Sci. USA.

[26] Sugimoto N, Maehara K, Yoshida K, Ohkawa Y, Fujita M (2018). Genome-wide analysis of the spatiotemporal regulation of firing and dormant replication origins in human cells. Nucleic Acids Res..

[27] Takahashi T, Ohara E, Nishitani H, Masukata H (2003). Multiple ORC-binding sites are required for efficient MCM loading and origin firing in fission yeast. EMBO J..

[28] Chambers VS (2015). High-throughput sequencing of DNA G-quadruplex structures in the human genome. Nat. Biotechnol..

[29] Bedrat A, Lacroix L, Mergny JL (2016). Re-evaluation of G-quadruplex propensity with G4Hunter. Nucleic Acids Res..

[30] Mehanna A, Diffley JF (2012). Pre-replicative complex assembly with purified proteins. Methods.

[31] Fernandez-Cid A (2013). An ORC/Cdc6/MCM2-7 complex is formed in a multistep reaction to serve as a platform for MCM double-hexamer assembly. Mol. Cell.

[32] Samel SA (2014). A unique DNA entry gate serves for regulated loading of the eukaryotic replicative helicase MCM2-7 onto DNA. Genes Dev..

[33] Coster G, Diffley JFX (2017). Bidirectional eukaryotic DNA replication is established by quasi-symmetrical helicase loading. Science.

[34] Pridgeon, C. & Corne, D. Hypermotifs: Novel discriminatory patterns for nucleotide sequences and their application to core promoter prediction in eukaryotes. In *Proc. 2005 IEEE Symposium on Computational Intelligence in Bioinformatics and Computational Biology*. 420–426 (IEEE, 2005).

[35] Massip F (2019). Evolution of replication origins in vertebrate genomes: rapid turnover despite selective constraints. Nucleic Acids Res..

[36] Danis E (2004). Specification of a DNA replication origin by a transcription complex. Nat. Cell Biol..

[37] Valenzuela MS (2011). Preferential localization of human origins of DNA replication at the 5′-ends of expressed genes and at evolutionarily conserved DNA sequences. PLoS ONE.

[38] Karnani N, Taylor CM, Malhotra A, Dutta A (2010). Genomic study of replication initiation in human chromosomes reveals the influence of transcription regulation and chromatin structure on origin selection. Mol. Biol. Cell.

[39] Mesner LD (2011). Bubble-chip analysis of human origin distributions demonstrates on a genomic scale significant clustering into zones and significant association with transcription. Genome Res..

[40] Cadoret JC (2008). Genome-wide studies highlight indirect links between human replication origins and gene regulation. Proc. Natl Acad. Sci. USA.

[41] McLean CY (2010). GREAT improves functional interpretation of cis-regulatory regions. Nat. Biotechnol..

[42] Claire Fonti, A. S. et al. Distinct oncogenes drive different genome and epigenome alterations in human mammary epithelial cells. *Int. J Cancer* **145**, 1299–1311. 10.1002/ijc.32413 31093963

[43] Dixon JR (2012). Topological domains in mammalian genomes identified by analysis of chromatin interactions. Nature.

[44] Foulk MS, Urban JM, Casella C, Gerbi SA (2015). Characterizing and controlling intrinsic biases of lambda exonuclease in nascent strand sequencing reveals phasing between nucleosomes and G-quadruplex motifs around a subset of human replication origins. Genome Res..

[45] Yeeles JT, Deegan TD, Janska A, Early A, Diffley JF (2015). Regulated eukaryotic DNA replication origin firing with purified proteins. Nature.

[46] Fragkos M, Ganier O, Coulombe P, Mechali M (2015). DNA replication origin activation in space and time. Nat. Rev. Mol. Cell Biol..

[47] Kolesnikova, S. & Curtis, E. A. Structure and function of multimeric G-quadruplexes. *Molecules*10.3390/molecules24173074 (2019). 10.3390/molecules24173074 PMC6749722 31450559

[48] Prorok P (2019). Involvement of G-quadruplex regions in mammalian replication origin activity. Nat. Commun..

[49] Shin G, Jeong D, Kim H, Im JS, Lee JK (2019). RecQL4 tethering on the pre-replicative complex induces unscheduled origin activation and replication stress in human cells. J. Biol. Chem..

[50] Sangrithi MN (2005). Initiation of DNA replication requires the RECQL4 protein mutated in Rothmund-Thomson syndrome. Cell.

[51] Moriyama K, Yoshizawa-Sugata N, Masai H (2018). Oligomer formation and G-quadruplex binding by purified murine Rif1 protein, a key organizer of higher-order chromatin architecture. J. Biol. Chem..

[52] Kliszczak M (2015). Interaction of RECQ4 and MCM10 is important for efficient DNA replication origin firing in human cells. Oncotarget.

[53] Keller H (2014). The intrinsically disordered amino-terminal region of human RecQL4: multiple DNA-binding domains confer annealing, strand exchange and G4 DNA binding. Nucleic Acids Res..

[54] Valton AL (2014). G4 motifs affect origin positioning and efficiency in two vertebrate replicators. EMBO J..

[55] Liu X, Kraus WL, Bai X (2015). Ready, pause, go: regulation of RNA polymerase II pausing and release by cellular signaling pathways. Trends Biochem. Sci..

[56] Cheneby J, Gheorghe M, Artufel M, Mathelier A, Ballester B (2018). ReMap 2018: an updated atlas of regulatory regions from an integrative analysis of DNA-binding ChIP-seq experiments. Nucleic Acids Res..

[57] Wang X (2017). Targeting of polycomb repressive complex 2 to RNA by short repeats of consecutive guanines. Mol. Cell.

[58] Beltran M (2019). G-tract RNA removes Polycomb repressive complex 2 from genes. Nat. Struct. Mol. Biol..

[59] Macheret M, Halazonetis TD (2018). Intragenic origins due to short G1 phases underlie oncogene-induced DNA replication stress. Nature.

[60] Barbet R, Peiffer I, Hatzfeld A, Charbord P, Hatzfeld JA (2011). Comparison of gene expression in human embryonic stem cells, hESC-derived mesenchymal stem cells and human mesenchymal stem cells. Stem Cells Int..

[61] Oburoglu L (2014). Glucose and glutamine metabolism regulate human hematopoietic stem cell lineage specification. Cell Stem Cell.

[62] Rodriguez-Martinez M (2017). The gastrula transition reorganizes replication-origin selection in Caenorhabditis elegans. Nat. Struct. Mol. Biol..

[63] Zhang Y (2008). Model-based analysis of ChIP-Seq (MACS). Genome Biol..

[64] Xu S, Grullon S, Ge K, Peng W (2014). Spatial clustering for identification of ChIP-enriched regions (SICER) to map regions of histone methylation patterns in embryonic stem cells. Methods Mol. Biol..

[65] Heinz S (2010). Simple combinations of lineage-determining transcription factors prime cis-regulatory elements required for macrophage and B cell identities. Mol. Cell.

[66] Zeng, Y. & Yi, C. sparseSVM: Fit sparse linear SVM with lasso or elasti-net regularization (2018).

[67] Pope BD (2014). Topologically associating domains are stable units of replication-timing regulation. Nature.

