# Supplementary material for: A predictable conserved DNA base composition signature defines human core DNA replication origins
Source: Nat Commun. 2020 Sep 21;11:4826. doi: 10.1038/s41467-020-18527-0 (PMC7506530; doi:10.1038/s41467-020-18527-0)
Supplement: Supplementary file 1 — Supplementary Information [file 41467_2020_18527_MOESM1_ESM.pdf]

A predictable conserved DNA base composition signature defines human core DNA replication origins

Akerman et al.

Supplementary Figures

Supplementary Figure1

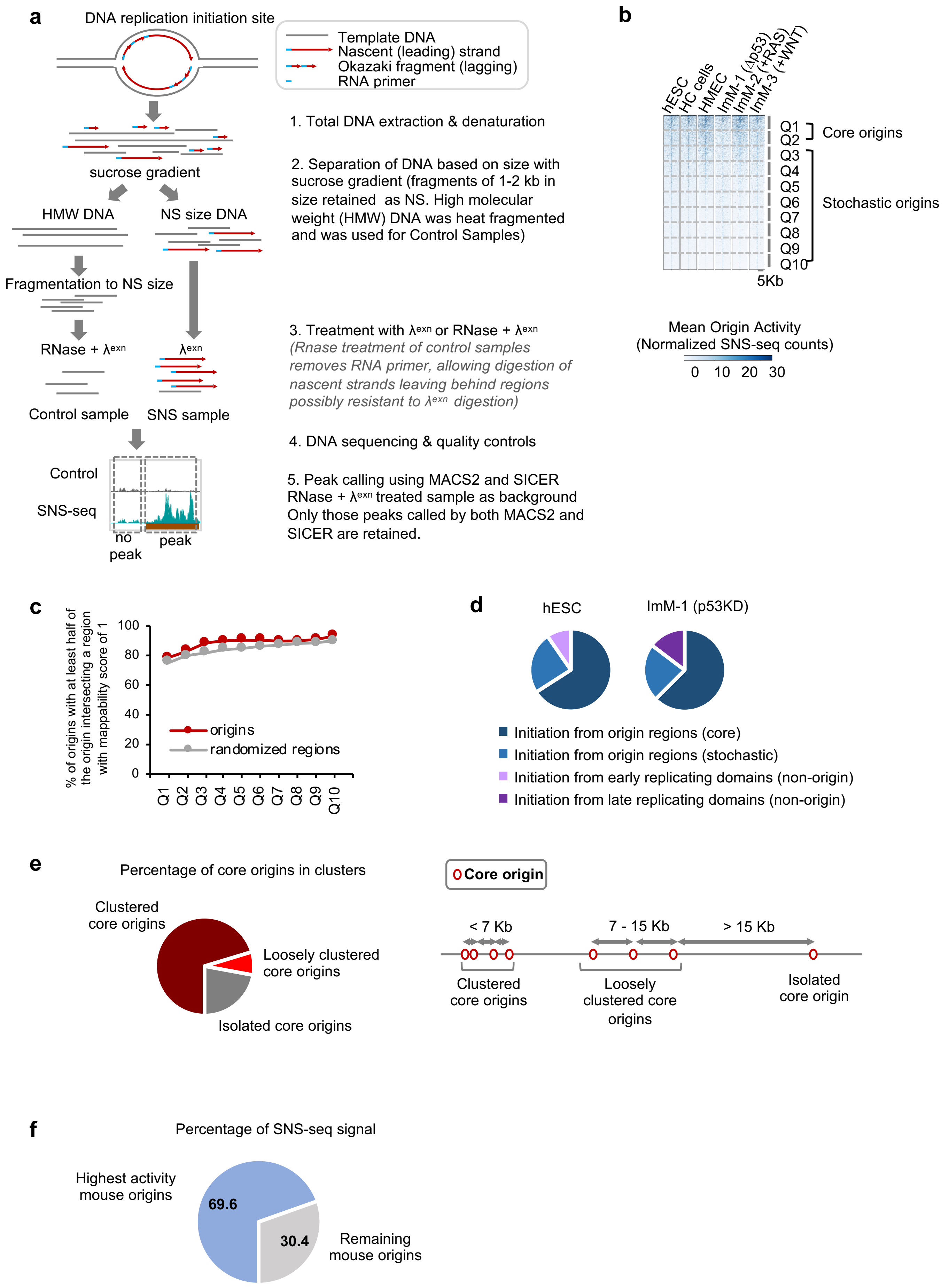

Supplementary Figure 1. Human origin repertoire

(**a**) Summary of the experimental SNS-seq procedure with the appropriate controls.
(**b**) Origin activity heatmap of all the identified human origins in six different cell lines. Origins were sorted according to their average activity based on the number of normalized SNS-seq reads. Human origins were then divided in ten equal-size quantiles (Q1-Q10) that included 32,074 origins/each.
(**c**) Mappability is similar for origins across different quantiles. Percentage of origins in each quantile with at least 50% of the origin overlapping fully mappable regions (UCSC-Umap, mappability score of 1).
(**d**) Broad and diffuse initiation outside the mapped origin regions is not substantial. Analysis of total diffuse initiation in early and late replicating domains of the human genome reveals that only two cell types have some initiation signal outside origin regions. In hESC cells. 9.6% of all DNA replication initiation comes from early (but not late) replicating domains outside the identified origin regions. Im ImM-1 cell type, 14.7% of all initiation comes from late-replicating (but not early replicating) domains, outside the origin regions.
(**e**) Most core origins are clustered in the genome. Pie chart showing the percentage of core origins found (i) clustered (i.e., less than 7 kb from each other), (ii) loosely clustered (more than 7 kb, but less than 15 kb from each other), and (iii) isolated (more than 15kb to the nearest core origin). Right panel depicts a schematic of the different clusters defined.
(**f**) A similar number of regions in the mouse genome also host the bulk of DNA replication initiation events. Pie chart showing the percentage of normalized SNS-seq tags that include the most active 64,148 origins (same number as in human cells) and the remaining lower activity origins.

Supplementary Figure2

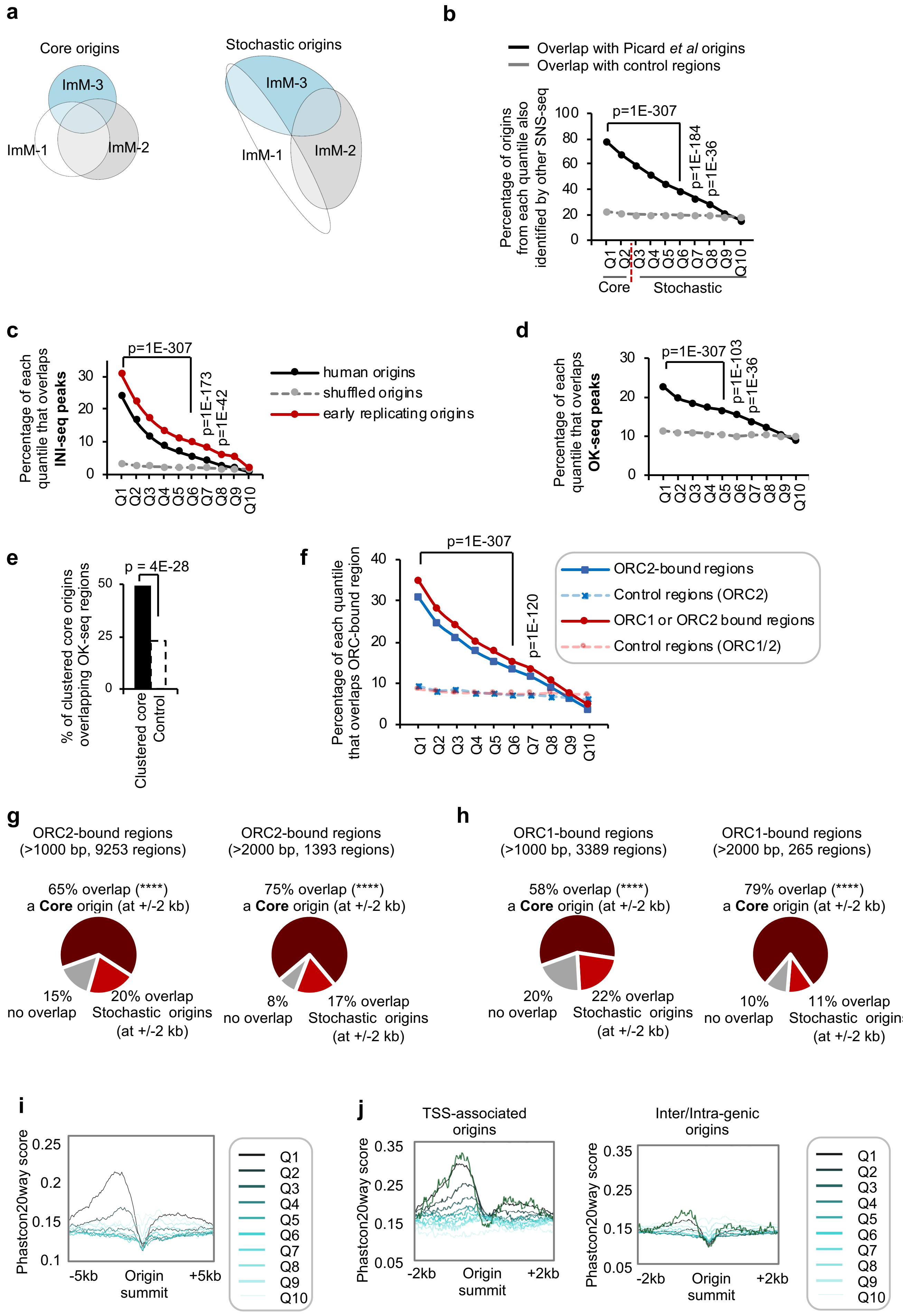

Supplementary Figure 2. Higher activity origins display higher ubiquity across replicates and cell types

(**a**) Euler diagrams showing the fraction of origins shared by three immortalized cell lines.

(**b**) Black dots show the percentage of origins in each quantile that overlap origins detected in a previous SNS-seq[1] study. Grey dots represent the expected chance overlaps of randomly shuffled, control genomic regions of equal size and number as our origins. P-values obtained by Chi-square Goodness-of-Fit test using observed and expected values for overlap.

(**c**) As in (b) for regions identified by INI-seq[2]. Red dots depict the percentage of early-firing origins identified by INI-seq[2], which is an *in vitro* method that identifies earliest firing origins.

(**d**) As in (b) for OK-seq[3] regions.

(**e**) Tightly clustered core origins are more likely to be identified by the alternative origin mapping method OK-seq[3]. Bar plot showing the percentage of tightly clustered core origins (in black) that overlap with DNA replication initiation zones identified by OK-seq. Dotted bars represent the expected chance overlap of randomly shuffled, control genomic regions of equal size and number to OK-seq regions. P-values obtained by Chi-square Goodness-of-Fit test using observed and expected values for overlap.

(**f**) Core origins overlap with the pre-RC components ORC1 and ORC2 binding sites. Graph shows the percentage of origins in each quantile that overlap with regions bound by ORC1 or ORC2 (red) or ORC2 (blue) within ± 2 kb. Paler coloured dots represent the expected chance overlap of randomly shuffled, control genomic regions of equal size and number as our origins.

(**g**) ORC2 binding sites that occupy larger genomic regions are more likely to be associated with DNA replication origins. Pie chart represents the percentage of ORC2-bound sites in the genome that intersect a core or a stochastic origin (within ± 2Kb). Left panel represents ORC2-bound regions longer than 1Kb, and the right panel represents ORC2-bound regions longer than 2 Kb. p-values were obtained using the Chi-square of Goodness-of-Fit test in R with observed and expected overlap values.

(**h**) Same as in (g) for ORC1-bound regions.

(**i**) Core origins (Q1 and Q2) have conserved sequences upstream of the initiation site. Graph represents averaged Phastcon20scores of human origins (Q1-Q10), centered on the origin summit with flanking regions on each side. Origins are oriented to have the G-rich regions upstream.

(**j**) As depicted in (i) for origins that are associated or not associated with a TSS within +/- 2Kb

Supplementary Figure3

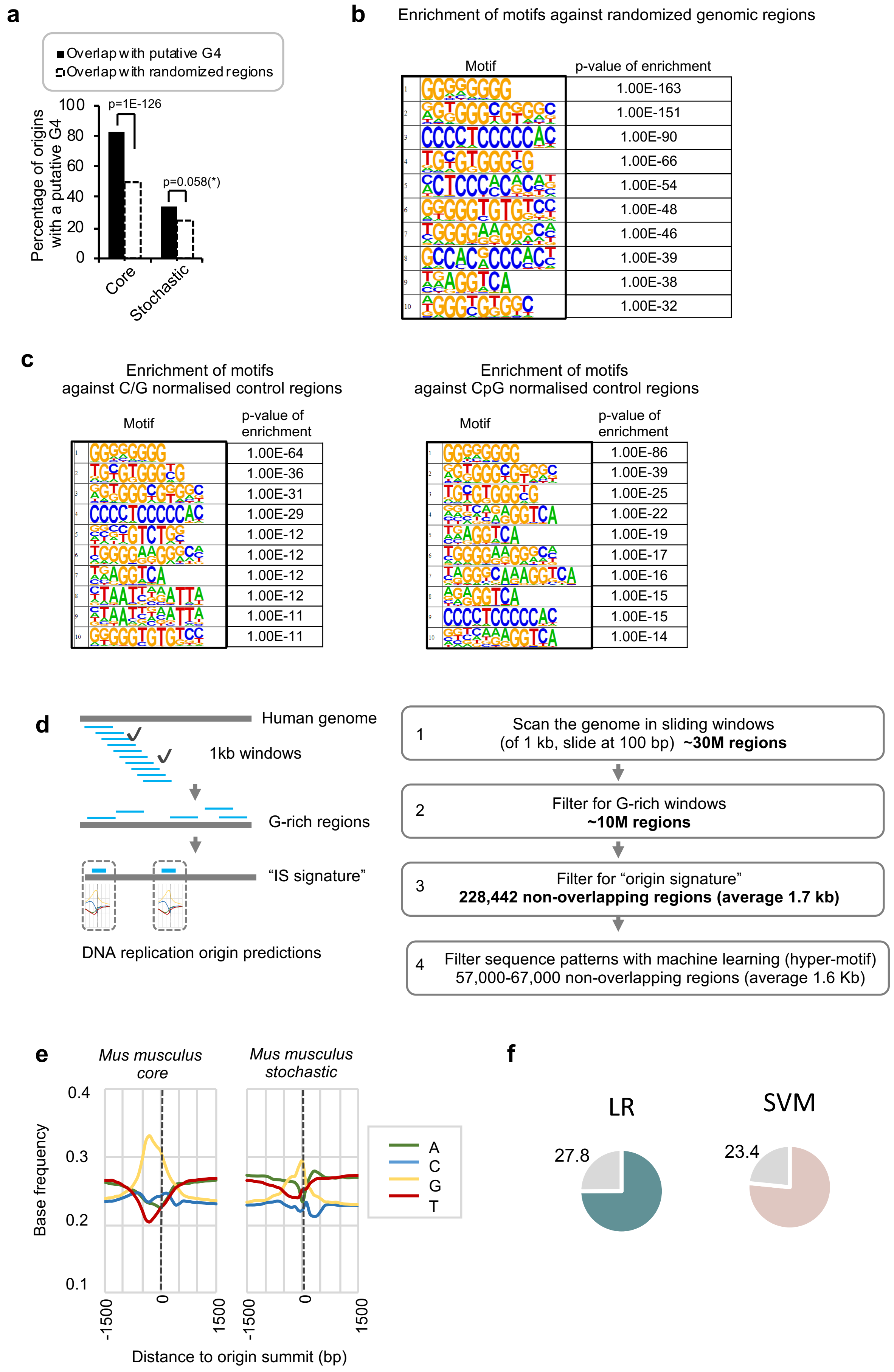

Supplementary Figure3 (continued)

g

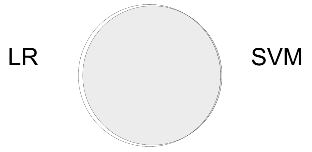

h

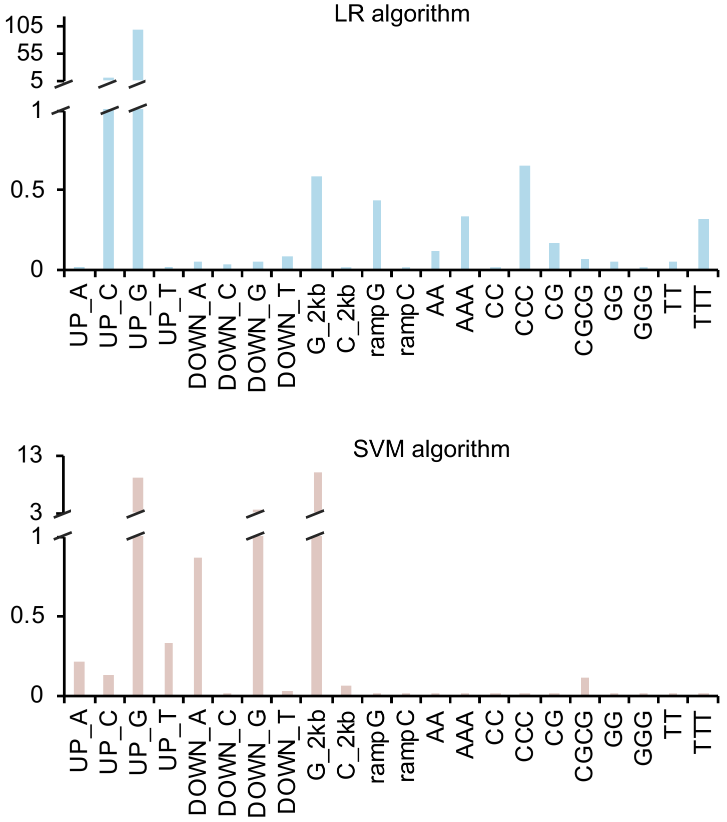

Supplementary Figure 3. The DNA sequence content is a major predictor of DNA replication IS
a) Bar plot representing the percentage of core and stochastic origins that overlap a putative G4 structure (in black) as defined by any one of the two methods used to define G4 structures (mismatch scoring[4] or G4Hunter[5]). Dotted lines represent expected overlaps with control regions, which are randomized regions of the genome of equal size and number to our origin regions. P-values represent Chi-square Goodness-of-Fit test using observed and expected values for overlap. (*) Please note that stochastic origins Q3-7 significantly overlap G4 regions (maximum p=0.0002) while Q8-10 do not.
(b) Motif enrichment analysis (using HOMER) for the regions covering 400 bp upstream of oriented core origins summits. Analysis in this figure represents enrichment over randomized genomic regions.
(**c**) Left panel represents motif enrichment over randomized genomic regions that contain the same C and G frequency as core origins. Right panel represents motif enrichment over randomized genomic regions that contain the same frequency of the dinucleotide "CG".
(d) Schematic diagram of the algorithm used to predict origins based on a DNA hyper-motif.
(**e**) Base content of the regions flanking mouse DNA replication (core and stochastic) origins and control genomic regions. Frequency plots are centred at the origin summits (highest point of the peak in a read pile-up). The base frequency represents the proportion of each base in sliding windows of 100 bp, on a scale from 0 to 1. Origins are oriented to have the side with the highest G-content upstream (see Methods for details).
(**f**) False positive rates (in gray) for three different machine learning algorithm methods. LR represents logistic regression with greedy feature selection, SVM represents univariate feature selection and single vector machine and uLR represents logistic regression with univariate feature selection.
(**g**) Different machine learning methods predict virtually the same core origins. Eulerr diagram (drawn to size) showing the overlap of core origins predicted by each machine learning method.
(**h**) The importance of each of the 22 features used for each machine learning algorithm. Top panel represents the weights assigned to each feature by the LR algorithm (in blue). Bottom panel represents the weights assigned to each feature by the SVM algorithm (in pink). The detailed explanation of each feature (x-axis) can be found in Table 2. Y-axis is of arbitrary units representing the importance assigned to each variable by each algorithm.

Supplementary Figure4

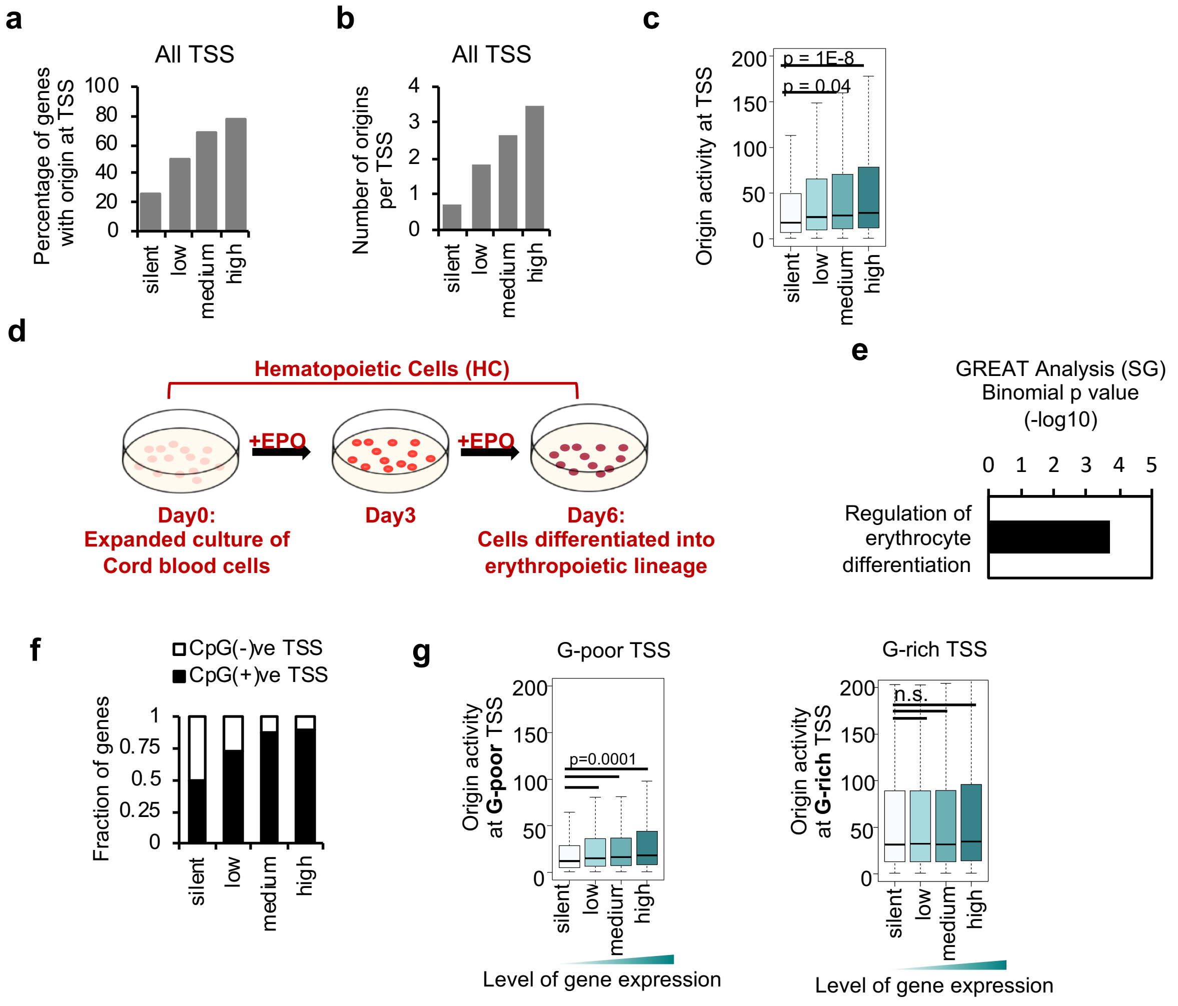

Supplementary Figure 4. Relationship between origin activity and nearby transcription
(a) Bar plot representing percentage of all Gencode (v25) gene promoters that host a DNA replication origin within +/- 2Kb of their TSS. Promoters with different transcriptional activity levels in hematopoietic cells are shown (silent = 0, low = 0-15, medium = 15-60, high = >60 RPKM).
(**b**) Bar plot showing the average number of origins localized within the promoter region (+/-2 Kb of the TSS) of genes with different transcriptional output levels (silent = 0, low = 0-15, medium = 15-60, high = >60 RPKM) in hematopoietic cells.
(**c**) Boxplots showing the average activity of origins localized in the promoter region (+/-2 Kb of the TSS) of genes with different transcriptional output levels as in (d) in hematopoietic cells. p-values were obtained using the Wilcoxon test in R. Line within the boxplot represents median, whereas the bounds of the box define the first and third quartiles. Bottom and top of whiskers represent minimum and maximum numbers respectively for each boxplot.
(**d**) Schematic summary of the hematopoietic cell (HC) differentiation protocol. HC (CD34+) were isolated from three independent human cord blood donors and expanded in three independent cultures for 6-7 days. Then, erythropoietin (+EPO) was added to the culture medium (Day 0) for 6 days, and cells were harvested at day 0, day 3 and day 6 for SNS-seq and RNA-seq analysis.
(**e**) Origins with increased activity after erythrocyte differentiation (day 6) are in genomic regions that host genes related to erythrocyte differentiation. The genomic coordinates of origins that were significantly upregulated upon EPO addition (day 0 vs day 6) were analysed with GREAT. GREAT analysis was performed on genomic coordinates of the origins that were significantly upregulated upon EPO treatment (day 0 vs day 6). Origin regions were associated with genes using the single-gene (SG) rule of GREAT. Only one category came up as statistically significant at Binomial p-value $p<0.05$, which was plotted here.
(**f**) Silent genes are less likely to contain a CpG island (CpGi) near their promoter region. Bar plots represent the fraction of GENCODE (v25) genes with different transcriptional activity levels in hematopoietic cells (defined as in a) that contain (CpG(+), in black) or not (CpG(-), in white) a CpGi within their TSS region (± 2Kb).
(**g**) Boxplots showing the average activity of origins localized within the promoter region (+/- 2 Kb of the TSS) of genes with different transcriptional outputs (silent = 0, low = 0-15, medium = 15-60, high = >60 RPKM). A G-rich TSS was defined as a TSS that contains a G-rich (>37% per 500 bp) stretch of DNA within ± 2Kb); p-values for significance in this figure are obtained using Wilcoxon test in R. Line within the boxplot represents median, whereas the bounds of the box define the first and third quartiles. Bottom and top of whiskers represent minimum and maximum numbers respectively for each boxplot.

Supplementary Figure5

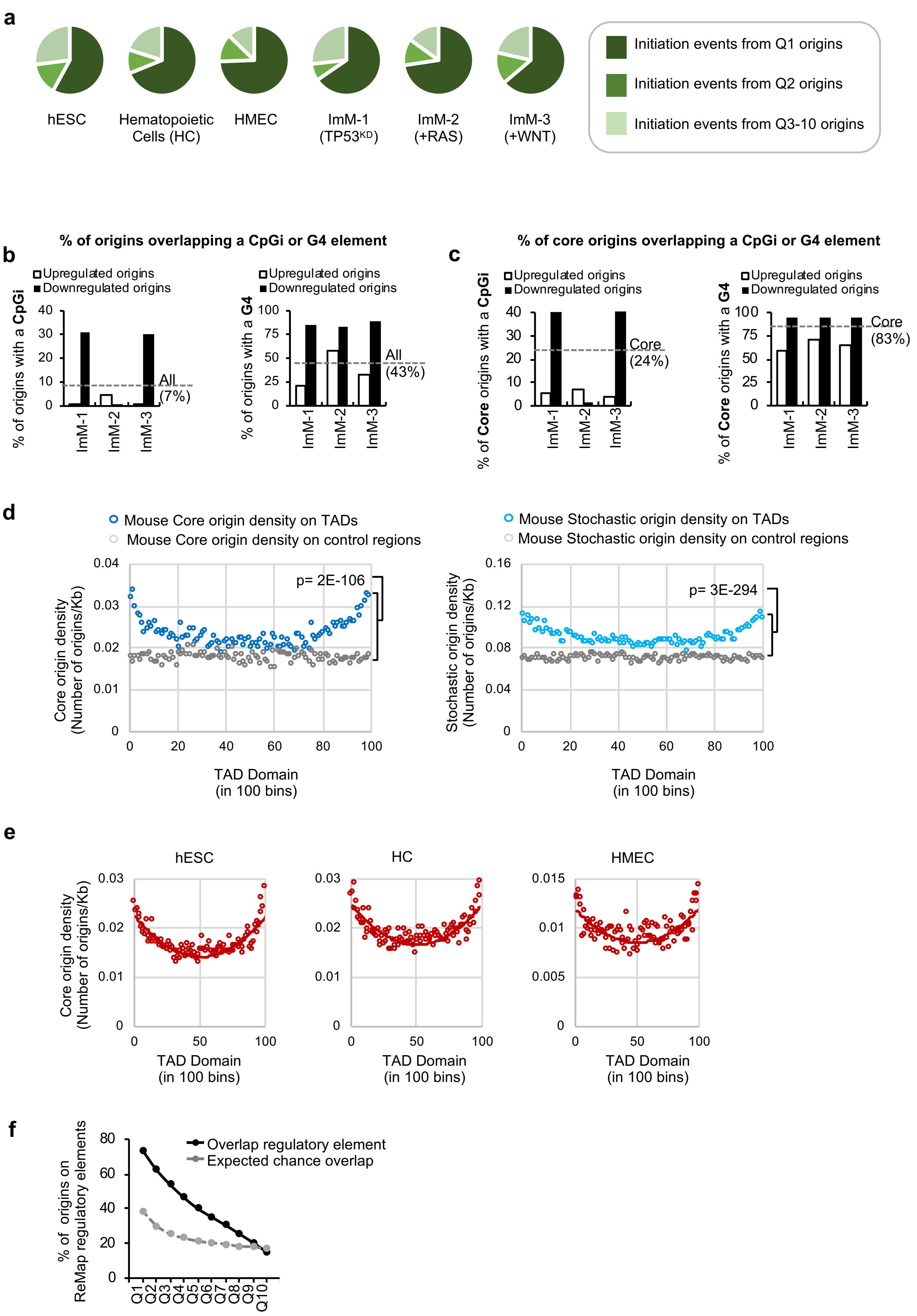

Supplementary Figure 5. Cell immortalization alters the DNA replication origin positioning within heterochromatin and TAD borders

(**a**) Pie charts representing the percentage of DNA replication initiation events (as assessed by normalized SNS-seq counts) at known origins that originate from Q1, Q2 (core origins) or Q3-10 (stochastic origins) in all cell types used in this study.

(**b**) Origin G-rich sequence-specificity is lost upon immortalization. In immortalized cells, origins that are down-regulated (black bars) in comparison to the parental cell line (HMEC) tend to overlap with CpGi (left panel) or G4 (right panel) elements. In contrast, origins upregulated upon immortalization (in white bars) have less than expected overlaps with CpGi or G4 elements. For reference, the dotted line shows the percentage of all origins that overlap with a CpGi (left panels) or G4 (right panels) are shown.

(**c**) Same as in (b), but for core origins that are up- or down-regulated upon immortalization. For reference, the dotted line shows the percentage of core origins that overlap with a CpGi (left panels) or G4 (right panels) are shown.

(**d**) Mouse core (left panel) and stochastic (right panel) origin density across topologically associating domains (TADs) of mouse embryonic stem cells[6]. Origin density along TAD domains (blue) or equal-size control regions (grey) was computed as follows. TADs were divided into 100 equal bins (slices) and the origin density in each bin was calculated as number of origins per Mb. The p-value was calculated using the non-parametric Wilcoxon test in R.

(**e**) Core origin density across TADs (determined in hESC H1) that are active in hESC H9 (left panel), HC (middle panel) or HMEC (right panel). Origin density along TADs was computed as in (d).

(**f**) Core origins coincide with putative regulatory elements. Plot shows the overlap of origins (Q1-Q10) with human genome regions that have putative regulatory functions (as defined by ReMap, >10 peaks).

References for Supplemental Information

1. Picard, F., Cadoret, J. C., Audit, B., Arneodo, A., Alberti, A., Battail, C., Duret, L. & Prioleau, M. N. The spatiotemporal program of DNA replication is associated with specific combinations of chromatin marks in human cells. *PLoS Genet* **10**, e1004282, doi:10.1371/journal.pgen.1004282 (2014).
2. Langley, A. R., Graf, S., Smith, J. C. & Krude, T. Genome-wide identification and characterisation of human DNA replication origins by initiation site sequencing (ini-seq). *Nucleic Acids Res* **44**, 10230-10247, doi:10.1093/nar/gkw760 (2016).
3. Petryk, N., Kahli, M., d'Aubenton-Carafa, Y., Jaszczyszyn, Y., Shen, Y., Silvain, M., Thermes, C., Chen, C. L. & Hyrien, O. Replication landscape of the human genome. *Nat Commun* **7**, 10208, doi:10.1038/ncomms10208 (2016).
4. Chambers, V. S., Marsico, G., Boutell, J. M., Di Antonio, M., Smith, G. P. & Balasubramanian, S. High-throughput sequencing of DNA G-quadruplex structures in the human genome. *Nat Biotechnol* **33**, 877-881, doi:10.1038/nbt.3295 (2015).
5. Bedrat, A., Lacroix, L. & Mergny, J. L. Re-evaluation of G-quadruplex propensity with G4Hunter. *Nucleic Acids Res* **44**, 1746-1759, doi:10.1093/nar/gkw006 (2016).
6. Dixon, J. R., Selvaraj, S., Yue, F., Kim, A., Li, Y., Shen, Y., Hu, M., Liu, J. S. & Ren, B. Topological domains in mammalian genomes identified by analysis of chromatin interactions. *Nature* **485**, 376-380, doi:10.1038/nature11082 (2012).
