# Supplementary material for: A predictable conserved DNA base composition signature defines human core DNA replication origins
Source: Nat Commun. 2020 Sep 21;11:4826. doi: 10.1038/s41467-020-18527-0 (PMC7506530; doi:10.1038/s41467-020-18527-0)
Supplement: Supplementary file 2 — Description of Additional Supplementary Files [file 41467_2020_18527_MOESM2_ESM.pdf]

Description of Additional Supplementary Files

File name: Supplementary Data 1
Description: This table includes the coordinates of called DNA replication origins, their properties, average activity levels across 19 samples and categorization (Q1-Q10, tissue specific).

File name: Supplementary Data 2
Description: This table includes predictor parameters and their description used for machine learning in this study.

File name: Supplementary Data 3
Description: Sources of datasets used in this study, including the name of the publication and the URL access.

File name: Supplementary Data 4
Description: List of primers and their sequences used in this study.

File name: Supplementary Data 5
Description: Confusion table displaying the performance of the genome scan (GS) and the machine learning algorithms on the test set.
