# Supplementary material for: A predictable conserved DNA base composition signature defines human core DNA replication origins
Source: Nat Commun. 2020 Sep 21;11:4826. doi: 10.1038/s41467-020-18527-0 (PMC7506530; doi:10.1038/s41467-020-18527-0)
Supplement: Supplementary file 8 — Reporting Summary [file 41467_2020_18527_MOESM8_ESM.pdf]

nature research

Corresponding author(s): Ildem Akerman and Marcel Mechali

Last updated by author(s): July 14, 2020

Reporting Summary

Nature Research wishes to improve the reproducibility of the work that we publish. This form provides structure for consistency and transparency in reporting. For further information on Nature Research policies, see Authors & Referees and the Editorial Policy Checklist.

Statistics

For all statistical analyses, confirm that the following items are present in the figure legend, table legend, main text, or Methods section.

n/a	Confirmed	
☒	☐	The exact sample size (n) for each experimental group/condition, given as a discrete number and unit of measurement
☒	☐	A statement on whether measurements were taken from distinct samples or whether the same sample was measured repeatedly
☐	☒	The statistical test(s) used AND whether they are one- or two-sided<br>*Only common tests should be described solely by name; describe more complex techniques in the Methods section.*
☒	☐	A description of all covariates tested
☒	☐	A description of any assumptions or corrections, such as tests of normality and adjustment for multiple comparisons
☐	☒	A full description of the statistical parameters including central tendency (e.g. means) or other basic estimates (e.g. regression coefficient) AND variation (e.g. standard deviation) or associated estimates of uncertainty (e.g. confidence intervals)
☐	☒	For null hypothesis testing, the test statistic (e.g. F, t, r) with confidence intervals, effect sizes, degrees of freedom and P value noted<br>*Give P values as exact values whenever suitable.*
☒	☐	For Bayesian analysis, information on the choice of priors and Markov chain Monte Carlo settings
☒	☐	For hierarchical and complex designs, identification of the appropriate level for tests and full reporting of outcomes
☐	☒	Estimates of effect sizes (e.g. Cohen's d, Pearson's r), indicating how they were calculated

Our web collection on statistics for biologists contains articles on many of the points above.

Software and code

Policy information about availability of computer code

Data collection	Data collection (for illumina sequencing) was performed by MGX facility, IGH, France, using bcl2fastq version 2.17 to generate fastq files.
Data analysis	A number of open source code was used to analyze this data as well as custom code.<br>FastQC 0.11.5<br>Bowtie 2.2.6 and 2.2.8<br>MACS2 2.2.1<br>SICER v1.1 (modified to contain the human hg38 genome)<br>bedtools 2.25<br>samtools 1.8<br>Rstudio and R (both versions 3.2, 3.4 and 3.5 used - since some packages are specific to a certain version)<br>on R, we have used: Diffbind (3.9), Deseq2 (1.18.0 and 1.2.0), ggplot2 (3.1.0), pheatmap (1.0.12)<br>edgeR 3.8.6<br>TopHat 2.1.1<br>HT-seq 0.61p1<br>UCSC toolkit (no version available)<br>HOMER (v4.11.1)<br>We have used CaRROT and SVM software for machine learning analysis- details indicated in Methods (open course on R).

For manuscripts utilizing custom algorithms or software that are central to the research but not yet described in published literature, software must be made available to editors/reviewers. We strongly encourage code deposition in a community repository (e.g. GitHub). See the Nature Research guidelines for submitting code & software for further information.

Data

Policy information about availability of data

All manuscripts must include a data availability statement. This statement should provide the following information, where applicable:

- Accession codes, unique identifiers, or web links for publicly available datasets
- A list of figures that have associated raw data
- A description of any restrictions on data availability

Data availability
Gene expressino omnibus database:
accession number: GSE128477 (released to public on 23rd July 2020)

Code availability (open access)
Github: https://github.com/iakerman/SNS-seq.

Field-specific reporting

Please select the one below that is the best fit for your research. If you are not sure, read the appropriate sections before making your selection.

☒ Life sciences ☐ Behavioural & social sciences ☐ Ecological, evolutionary & environmental sciences

For a reference copy of the document with all sections, see nature.com/documents/nr-reporting-summary-flat.pdf

Life sciences study design

All studies must disclose on these points even when the disclosure is negative.

Sample size	Our total sample size for human samples is 22, while it is 9 for mouse cell types.<br>For each experimental condition (i.e. differentiation stage, or cell type) we have three experimental replicates (n=3) except for hESC and +WNT conditions, where we have n=2.<br>SNS-seq analysis is most similar to ChIP-seq analysis in nature and the ENCODE standards for ChIP-seq analysis dictates that we use at least n=2 for statistically sound analysis. Thus, we abide by the ENCODE standards (https://www.encodeproject.org/about/experiment-guidelines/)
Data exclusions	One SNS-seq sample was excluded based on sequence quality, most likely due to failed library preparation. Another RNA-seq sample was excluded, due to an error in library preparation (sequencing failure).
Replication	Since the initial submission of this paper, we have obtained additional mouse SNS-seq samples (Prorok et al Nature Comm 2019). and have confirmed the presence of core origins in these new samples.<br>Core origins were also observed in the data of an independent study by Li lab:<br>Long, H. et al. H2A.Z facilitates licensing and activation of early replication origins. Nature 577, 576-581 (2020).
Randomization	While we have control samples in our study (control SNS-seq samples), out study does not involve any clinical trials or treatments on patients/individuals.
Blinding	Since the experiments involve differential treatment of samples (control vs experimental) it was not possible to blind the experimenter.

Reporting for specific materials, systems and methods

We require information from authors about some types of materials, experimental systems and methods used in many studies. Here, indicate whether each material, system or method listed is relevant to your study. If you are not sure if a list item applies to your research, read the appropriate section before selecting a response.

Materials & experimental systems

n/a	Involved in the study	
☒	☐	Antibodies
☐	☒	Eukaryotic cell lines
☒	☐	Palaeontology
☒	☐	Animals and other organisms
☐	☒	Human research participants
☒	☐	Clinical data

Methods

n/a	Involved in the study	
☒	☐	ChIP-seq
☒	☐	Flow cytometry
☒	☐	MRI-based neuroimaging

Eukaryotic cell lines

Policy information about cell lines

Cell line source(s)	hESC were from ESI, Singapore. Other cell types were either primary or generated by our collaborators as described in materials and methods.<br>Hematopoietic cells: CD34+ cells were isolated from umbilical cord blood obtained following delivery of deidentified full-term infants and treated with erythropoietinin (EPO) for the indicated durations. These cells were generated by Naomi Taylor Lab.<br>HMEC, p53knockdown, +WNT and +RAS cell lines were generaetd by the Charles Thelliet laboratory through over-expression or knockdown of the indicated proteins (see manuscript).<br>HMEC cells are primary cell lines, initially generated by Charles Thelliet lab.
Authentication	For the in-house generated lines (hematopoietic), we used quantitative PCR to confirm the identity of the CD34+ isolated progenitors as well differentiated cells using markers. We use a combination of 6+ markers that are in routine use by the Dr. Naomi Taylor Lab.<br>HMEC cells and derivatives were generated and characterized by Charles Theillet lab as described (see Methods).<br>Finally, hESC are routinely screened for the expression of stem cell markers (such as OCT4). hESC are maintained by a trained technician.
Mycoplasma contamination	Cells are routinely checked for contamination- any contaminated cells are removed immediately.
Commonly misidentified lines<br>(See ICLAC register)	none.

Human research participants

Policy information about studies involving human research participants

Population characteristics	The samples were obtained from umbilical cord blood following delivery of deidentified full-term infants (age = day 1). However no further information regarding the individual was kept.
Recruitment	CD34+ cells were isolated from umbilical cord blood obtained following delivery of deidentified full-term infants after written informed consent from the mothers at the University Hospital of Montpellier, France.
Ethics oversight	Use of these deidentified samples was determined to be exempt from ethical review by the University Hospital of Montpellier Institutional Review Board in accordance with the guidelines issued by the Office of Human Research Protections.

Note that full information on the approval of the study protocol must also be provided in the manuscript.
